# Interleukin-23 instructs protective multifunctional CD4 T cell responses after immunization with the *Mycobacterium tuberculosis* subunit vaccine H1 DDA/TDB independently of interleukin-17A

**DOI:** 10.1007/s00109-021-02100-3

**Published:** 2021-08-05

**Authors:** Kristina Ritter, Jochen Behrends, Hanna Erdmann, Jasmin Rousseau, Alexandra Hölscher, Johanna Volz, Immo Prinz, Thomas Lindenstrøm, Christoph Hölscher

**Affiliations:** 1 Infection Immunology, Research Center Borstel, Borstel, Germany; 2 Fluorescence Cytometry Core Unit, Research Center Borstel, Borstel, Germany; 3 Institute of Immunology, Hannover Medical School, Hannover, Germany; 4 Center for Molecular Neurobiology Hamburg, Eppendorf University Medical Center, Hamburg, Germany; 5 Department of Infectious Disease Immunology, Statens Serum Institut, Copenhagen, Denmark

**Keywords:** Tuberculosis, Mice, Vaccination, Cytokines, T cells

## Abstract

**Abstract:**

Interleukin (IL)-17A-producing T helper (Th)17 cells are increasingly being acknowledged to be associated with protective immunity to *Mycobacterium tuberculosis* (Mtb). Subunit vaccines potently promote protective immune responses against Mtb infection that correlate with an expansion of IL-23-dependent Th17 cells. Previous studies revealed that after vaccination, IL-23 is required for protection against challenge with Mtb but the underlying IL-23-dependent—and possibly IL-17A-mediated—mechanisms remain elusive. Therefore, we here analyzed the early outcome of Mtb infection in C57BL/6, IL-23p19-deficient (^−/−^), and IL-17A^−/−^ mice after vaccination with the subunit vaccine H1-DDA/TDB to investigate the role of the IL-23-Th17 immune axis for the instruction of vaccine-induced protection. While in IL-23p19^−/−^ mice the protective effect was reduced, protection after vaccination was maintained in IL-17A^−/−^ animals for the course of infection of 6 weeks, indicating that after vaccination with H1-DDA/TDB early protection against Mtb is—although dependent on IL-23—not mediated by IL-17A. In contrast, IL-17A deficiency appears to have an impact on maintaining long-term protection. In fact, IL-23 instructed the vaccine-induced memory immunity in the lung, in particular the sustained expansion of tumor necrosis factor (TNF)^+^IL-2^+^ multifunctional T cells, independently of IL-17A. Altogether, a targeted induction of IL-23 during vaccination against Mtb might improve the magnitude and quality of vaccine-induced memory immune responses.

**Key messages:**

- After subunit Mtb vaccination with H1-DDA/TDB, IL-23 but not IL-17A contributes to vaccine-induced early protection against infection with Mtb.
- IL-17F does not compensate for IL-17A deficiency in terms of H1-DDA/TDB-induced protection against Mtb infection.
- IL 23 promotes the H1-DDA/TDB-induced accumulation of effector memory T cells independently of IL 17A.
- IL-23 arbitrates the induction of H1-specific IFN-γ^−^TNF^+^IL-2^+^ double-positive multifunctional CD4 T cells after subunit Mtb vaccination in an IL-17A-independent manner.

**Graphical abstract:**

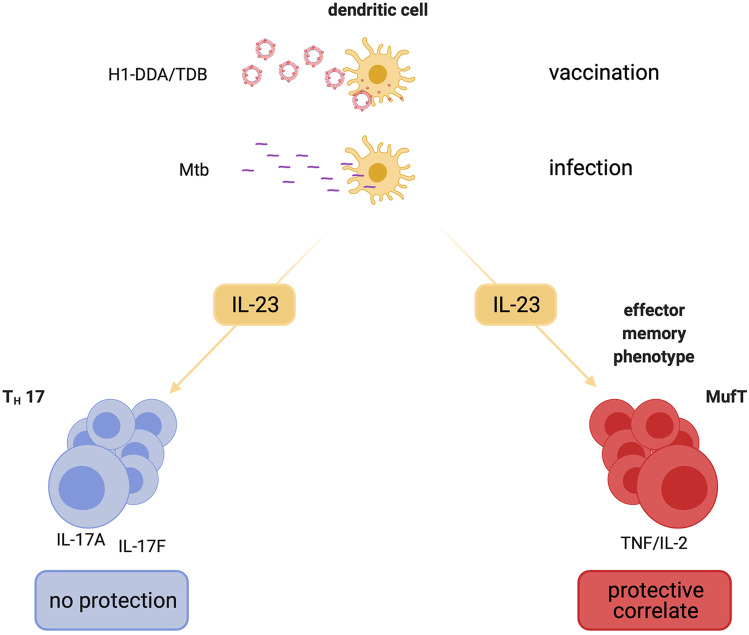

**Supplementary information:**

The online version contains supplementary material available at 10.1007/s00109-021-02100-3.

## Introduction

Tuberculosis (TB) remains one of the most serious health threats worldwide. Each year, *Mycobacterium tuberculosis* (Mtb), the causative agent of human TB, infects above 10 million people and causes almost 2 million deaths [1]. Effective vaccination against Mtb would therefore make an important contribution to global health. However, the only licensed TB vaccine, *Mycobacterium bovis* Bacillus Calmette-Guérin (BCG), provides insufficient protection against pulmonary TB in adults, the most common form of active disease [2]. Although over the past decades research on novel vaccination strategies against TB generated a range of promising candidates [3], a more complete knowledge about the immunological mechanisms of vaccine-mediated protection is still of critical importance.

CD4^+^ T cells, in particular interferon-γ (IFN-γ)-producing T helper type 1 (Th1) cells, play a central role in host resistance to primary infection with Mtb. Accordingly, mice lacking CD4, IFN-γ, or the Th1*-*promoting cytokine interleukin (IL)-12 are highly susceptible to Mtb infection [4-7]. In this regard, IFN-γ stimulates the antimicrobial activity of infected macrophages leading to the production of reactive nitrogen intermediates and eventually to intracellular elimination of bacteria [6, 7]. However, both animal models and human trials meanwhile revealed that the magnitude of IFN-γ production does not provide a reliable correlate of protection after vaccination against Mtb [8-10], indicating the contribution of additional immunological factors to the vaccine-induced defense.

Long-term memory immunity forms the basis of effective vaccination. As T cell longevity requires the presence of IL-2 [11], it is not surprising that T cell subsets co-expressing IL-2 appear to be associated with vaccine-mediated protection against Mtb [12-14]. Thus, high-quality multifunctional T cells co-expressing IFN-γ, TNF, and IL-2 are induced by subunit vaccination as well as BCG [12]. In contrast to BCG, subunit vaccination alone also results in the accumulation of TNF^+^IL-2^+^ double-positive multifunctional T cells, which might function as a key memory pool ensuring long-term containment of Mtb [12, 13].

It is reasonable to assume that IL-17A-producing Th17 cells function as early mediators of vaccine-induced protection against Mtb. The heterodimeric cytokine IL-23 is required to stabilize the Th17 phenotype [15, 16]. IL-23 belongs to the IL-12 family of heterodimeric cytokines and is composed of the subunits IL-23p19 and IL-12p40, the latter of which IL-23 shares with IL-12 [17]. Mice deficient for IL-23p19 exhibit unaltered bacterial growth in the early phase of infection with Mtb [18, 19]; however, for long-term containment of Mtb IL-23 is required [18]. Notably, while IL-23 is currently considered primarily to be essential for the establishment of a Th17 immune response, it was initially described as a stimulator of a memory T cell response [17, 20].

IL-17A, the main effector cytokine of Th17 cells, plays a differential role during primary infection with Mtb. Whereas the outcome of a low-dose infection with the lab-adapted strain Mtb H37Rv is unaffected in mice deficient for IL-17A, the cytokine is required for control of a high dose infection with 1 × 10^3^ colony-forming units (CFU) H37Rv and of infection with the hypervirulent W/Beijing strain HN878 [21, 22].

Several vaccine candidates against Mtb potently induce Th17 immune responses. These include, among others, Mtb adjuvants such as the mycobacterial cell wall glycolipid Trehalose-6,6-dimycolate (TDM) and its synthetic analog Trehalose-6,6-dibehenate (TDB) [15, 23, 24] but also different mucosal vaccines [25-29]. Following subcutaneous vaccination with BCG, Th17 cells accumulate in the draining lymph nodes at the early stage of Mtb infection and are critical for the recruitment of vaccine-induced Th1 cells [30]. Whereas IL-23 is dispensable during primary infection with Mtb H37Rv [19], the expression of IL-23 is required for antimycobacterial protection after subunit vaccination with 6-kilodalton early secreted antigenic target protein (ESAT-6)_1–20_ adjuvanted by a combination of TDM, monophosphoryl lipid A (MPL), and dimethyl dioctadecylammonium bromide (DDA) [15]. This discrepancy points at a specific function of IL-23 during recall responses. In fact, vaccine-induced IL-23-dependent IL-17A-producing Th17 cells populate the lung early after infection with Mtb- and in turn induce C-X-C motif ligand (CXCL)9, CXCL10, and CXCL11 chemokine expression to promote the recruitment of Th1 cells to the lung [15]. By contrast, adoptive transfer of BCG-specific Th17 cells mediate protection against Mtb in the absence of IFN-γ [31]. IFN-γ-independent protection is also provided by mucosal vaccination with ESAT-6_1–20_ in combination with the mucosal adjuvant LT-IIb [27]. In the latter case, IL-17A appeared to be indeed required for vaccine-induced protection, as here vaccination had no effect on bacterial growth in IL-17A-deficient (^−/−^) mice. However, to our knowledge it has not yet been investigated whether the IL-23-IL-17A immune axis is also relevant for a parenteral vaccination.

For this reason, the present study compared the protective immune response in IL-17A^−/−^ and IL-23p19^−/−^ mice after parenteral subunit vaccination with the fusion protein H1 (Ag85B-ESAT-6) combined with the adjuvant system DDA/TDB (also referred to as CAF01) and subsequent Mtb infection. We here show that, in contrast to IL-23, IL-17A unexpectedly is not required for early protection against Mtb after parenteral vaccination. IL-23 in fact mediates early protection after vaccination independently of IL-17A by the instruction of vaccine-induced CD4^+^ T cell memory responses and of memory-associated TNF^+^IL-2^+^ multifunctional T cells.

## Methods

### Mice

IL-17A^−/−^ (MGI nomenclature: B6.129P2-*Il17a*^tm1Yiw^) [32] and IL-23p19^−/−^ (MGI nomenclature: B6.129S5-*Il23a*^tm1Lex^) [34] breeding pairs were kindly provided by Yoichiro Iwakura (University of Tokyo, Tokyo, Japan) and Nico Ghilardi (Genentech, South San Francisco, CA, USA), respectively. Breeding pairs of IL-17A/F^−/−^ mice (MGI nomenclature: B6.Cg-*Il17a*/*Il17f*^tm1.1Impr^ Thy1a) [35] were obtained from the Hannover Medical School (Hannover, Germany). All strains were on a C57BL/6 genetic background. These mice were bred at the Institute of Animal Breeding and Husbandry at the Christian-Albrechts-University (Kiel, Germany). C57BL/6 wild-type mice were purchased from Charles River. Both females and males were used in all experiments. The respective proportion of females was approximately the same for all mouse strains. When C57BL/6, IL-23p19^−/−^, and IL-17A^−/−^ mice were compared, the proportion was 48.3 ± 3.0%, 47.7 ± 3.1%, and 48.1 ± 2.6%, respectively. When C57BL/6, IL-17A^−/−^, and IL-17A/F^−/−^ mice were compared, 70.0 + 10.0%, 66.7 ± 13.3%, and 65.1 ± 15.0% were females, respectively. In each experiment, cohorts of C57BL/6, IL-17A^−/−^, and IL-23p19^−/−^ mice, or C57BL/6, IL-17A^−/−^, and IL-17A/F^−/−^ mice, respectively, were vaccinated and infected simultaneously. During infection experiments, mice were kept under barrier conditions in individually ventilated cages in the BSL 3 facility at the Research Center Borstel (Borstel, Germany). All animal experiments were performed according to the German animal protection laws and were approved by the Animal Research Ethics Board of the Ministry of Energy, Agriculture, the Environment, Nature and Digitalization (Kiel, Germany) (approval number 72–5/13 and 56–7/18). In total, 650 animals were used for the here described experiments.

### Immunization

Vaccination was performed by use of the subunit vaccine H1 in combination with the adjuvant system DDA/TDB (also referred to as CAF01). Both subunit vaccine and adjuvant system were kindly provided by the Statens Serum Institut, Copenhagen, Denmark. Mice were vaccinated subcutaneously by footpad injection 3 times, with a 2-week interval between injections. Each mouse was vaccinated with 2 µg of the vaccine antigen H1 emulsified in 100 µl DDA/TDB (50 µl per foot). In addition, control mice were injected with 50 µl PBS per footpad. Four weeks after the third injection, unvaccinated and vaccinated mice were infected with 152 ± 27 CFU/lung Mtb H37Rv as described below.

### Bacteria and infection

For infection experiments, mice were infected with Mtb H37Rv. Before infection of experimental animals, stock solutions of Mtb H37Rv were diluted in sterile distilled water and pulmonary infection was performed using an inhalation exposure system (Glas-Col, Terre-Haute, IN). To achieve an infectious dose of approximately 150 CFU/lung, mice were exposed for 40 min to an aerosol generated by nebulizing 6.5 ml of a suspension containing 10^7^ live bacteria. The inoculum size was checked 24 h post-infection by determining the bacterial load in the entire lung of infected mice. Across all experiments, animals were infected with 152 ± 27 CFU/lung.

### Colony enumeration assay

Bacterial loads in lungs were evaluated at different time points after infection with Mtb. Lungs from sacrificed animals were removed aseptically, weighed, and homogenized in PBS containing a proteinase inhibitor cocktail (Roche Diagnostics, Mannheim, Germany) using the FastPrep™ System (MP Biomedicals, Solon, USA). Tenfold serial dilutions of organ homogenates were plated onto Middlebrook 7H10 agar plates containing 5% glycerine (AppliChem, Darmstadt, Germany) and 10% heat-inactivated bovine serum (Biowest, Nuaillé, France). Inoculated plates were afterward incubated at 37 °C. Colonies on plates were counted after 21 days.

### Histology

The left lung lobe of each mouse was fixed in 4% buffered formalin, embedded in paraffin blocks, and sectioned on a microtome (2 μm). For visual analysis of histopathology, the lung sections were stained with hematoxylin/eosin using standard protocols.

### Preparation of single-cell suspensions from infected lungs

For flow cytometric analysis and ESAT6_1-20_-specific Elispot assays, single-cell suspensions of lungs were prepared from Mtb-infected mice at different time points. Lungs were perfused through the right ventricle with warm PBS. Once lungs appeared white, they were removed and sectioned. Dissected lung tissue was incubated in collagenase A (0.7 mg/ml, Roche Diagnostics, Mannheim, Germany) and DNase (30 µg/ml, Sigma) at 37 °C for 2 h. Digested lung tissue was gently disrupted by subsequent passage through a 100-µm pore size nylon cell strainer. Suspensions were depleted from remaining erythrocytes using hypotonic red cell lysis buffer. Recovered vital lung cells were diluted in complete Iscove’s-modified Dulbecco’s medium (IMDM; PAA) supplemented with 10% FCS, 1% L-glutamine (200 mM; Biochrom), and 1% penicillin/streptomycin (10,000 U/ml and 10,000 mg/ml; Biochrom) and counted using an automatic cell counter (ViCell®, Beckman Coulter, Krefeld, Germany).

### Flow cytometry

For flow cytometric analysis of surface markers, single-cell suspensions of lungs were incubated with a mixture containing anti-FcγRIII/II antibody (Biolegend) as well as mouse, rat, and hamster serum to block nonspecific binding. Cells were then incubated with optimal concentrations of the following specific antibodies against surface molecules: anti-CD90.2-APC-eFluor780, anti-CD127-PE-Cy7 (all from eBioscience), anti-CD44-FITC (Biolegend), anti-CD4-V500, anti-CD62L-APC, and anti-KLRG1-BV711 (all from BD Biosciences). For intracellular cytokine staining, 0.8 × 10^6^ cells were stimulated with plate-bound anti-CD3/anti-CD28 (each 5 µg/ml, BD Bioscience) or H1 (5 μg/ml) for 4.5 h in the presence of GolgiPlug™ (BD Biosciences). Cell were stained with optimal concentrations of anti-CD4-V500 (BD Biosciences), anti-CD44-FITC (Biolegend), and anti-CD90.2-APC-eFluor780 (eBioscience). Afterward cells were fixed and permeabilized with Cytofix/Cytoperm™ (BD Biosciences). Intracellularly accumulated cytokines were stained with anti-IFN-γ-PE (Biolegend) or anti-IFN-γ-V450 (BD Bioscience), respectively, anti-TNF-PE-Cy7 (Biolegend), anti-IL-17A-PerCP-Cy5.5 (eBioscience), and anti-IL-2-APC (BD Biosciences). Data were acquired on a FACSCanto™II (BD Bioscience) or on a LSRII (BD Bioscience) and analyzed with the FCS Express 5 Flow Cytometry software (DeNovo™ Software).

### ESAT6_1-20_-specific Elispot assays

Analysis of antigen-specific IL-17A- or IFN-γ-producing cells from infected lungs was performed using Elispot assay kits (R&D Systems and BD Biosciences, respectively). To enrich CD4^+^ T cells, single-cell suspensions were incubated with magnetic CD4 MicroBeads (Miltenyi, Bergisch Gladbach, Germany) and separated from other cells using the autoMACS® Pro Separator (Miltenyi). Separated CD4^+^ T cells were resuspended in (IMDM) supplemented with 10% FCS, 1% L-glutamine (200 mM; Biochrom), and 1% penicillin/streptomycin (10,000 U/ml and 10,000 mg/ml; Biochrom) and counted. Cells were seeded in wells of anti-IFN-γ- or anti-IL-17A-coated MultiScreen HTS-IP filter plates (1 × 10^5^ cells/well) and two-fold serial dilutions were made. As antigen presenting cells mitomycin-D-inactivated splenocytes from uninfected wild-type mice were used (1 × 10^6^ cells/well). Cells were stimulated with ESAT6_1–20_ (10 µg/ml, Research Center Borstel) and recombinant IL-2 (10 U/ml, Peprotech, Hamburg, Germany) in 5% CO_2_ at 37 °C. After 20 h anti-IFN-γ-coated plates were washed and a biotinylated anti-IFN-γ-antibody was used to detect the captured cytokine. Spots were enumerated using streptavidin-HRP and AEC solution as a substrate. After 24 h anti-IL-17A-coated plates were washed and a biotinylated anti-IL-17A-antibody was used to detect the captured cytokine. Spots were visualized using streptavidin-AP and BCIP/NBT solution as a substrate. Spots were automatically enumerated using an Elispot reader (EliSpot 04 XL; AID).

### Quantitative real-time PCR

Lung samples of uninfected and infected mice were homogenized in 4 M guanidinium-isothiocyanate buffer and total RNA was extracted by acid phenol extraction. cDNA was obtained using RevertAid H Minus M-MuLV reverse transcriptase (Fermentas) and random hexamer (Fermentas) as a primer. Quantitative PCR was conducted on a Light Cycler^®^ 480 Instrument (Roche Diagnostics). Data were analyzed employing the “Second Derivative Maximum Method” and “Standard Curve Method” using hypoxanthine–guanine phosphoribosyltransferase (*Hprt*) as a housekeeping gene to calculate the level of gene expression in relation to *Hprt*. The following primer and probe sets were employed: *Hprt*: sense 5′-TCC TCC TCA GAC CGC TTT T-3′, antisense 5′-CCT GGT TCA TCA TCG CTA ATC-3′, probe 5′-AGT CCA G-3′; *Cxcl9*: sense 5′-CTT TTC CTC TTG GGC ATC AT-3′, antisense 5′-GCA TCG TGC ATT CCT TAT CA-3′, probe 5′-CCT GGA GC-3′; *Cxcl10*: sense 5′-GCT GCC GTC ATT TTC TGC-3′, antisense 5′-TCT CAC TGG CCC GTC ATC-3′, probe 5′-CTG CTG GG-3′; *Cxcl11*: sense 5′-TCT GCA AAG AGA GAT CTC CAA A-3′, antisense 5′-CGC CCC TGT TTG AAC ATA AG-3′, probe 5′-AGG CAG AG-3′; *Ifng*: sense 5′-ATC TGG AGG AAC TGG CAA AA-3′, antisense 5′-TTC AAG ACT TCA AAG AGT CTG AGG TA-3′, probe 5′-CAG AGC CA-3′; *Il17a*: sense 5′-TGT GAA GGT CAA CCT CAA AGT CT-3′, antisense 5′-GAG GGA TAT CTA TCA GGG TCT TCA T-3′, probe 5′-GCT CCA GA-3′; and *Il17f*: sense 5′-CCC AGG AAG ACA TAC TTA GAA GAA A-3′, antisense 5′-CAA CAG TAG CAA AGA CTT GAC CA-3′, probe 5′-ATG GCT GC-3′.

### Statistical analysis

Wild-type and gene-deficient mice, either vaccinated or not vaccinated, were compared at different time points of infection. No randomization was used before each experiment, group allocations were not blinded, and confounders were not controlled. No criteria were set for excluding animals from the study. In the rare case of a technical problem, such as contamination, samples were excluded before further examination. In all other cases, single data points were not excluded from the analysis. Statistical analysis was performed using Prism 9 (GraphPad Software, San Diego, USA). Quantifiable data are expressed as the means of individual determinations and standard deviations. For the majority of experiments, a Mann–Whitney test or a two-way ANOVA with a Bonferroni post hoc test was used. For the comparison of more than two groups of only one variable, a Kruskal*–*Wallis test with Dunn’s multiple comparison test was used. For P values ≤ 0.05, results were considered statistically significant. Because of the number of hypotheses to be tested, this study was regarded as purely exploratory. For this reason, the statistical tests and the resulting p-values can only be considered as descriptive and not interpreted as confirmatory. Unless otherwise indicated, data obtained from repeat experiments are presented in supplemental Fig. S3.

## Results

### In contrast to IL-17A, IL-23 promotes protection after vaccination with H1-DDA/TDB during Mtb infection

**Fig. 1:**
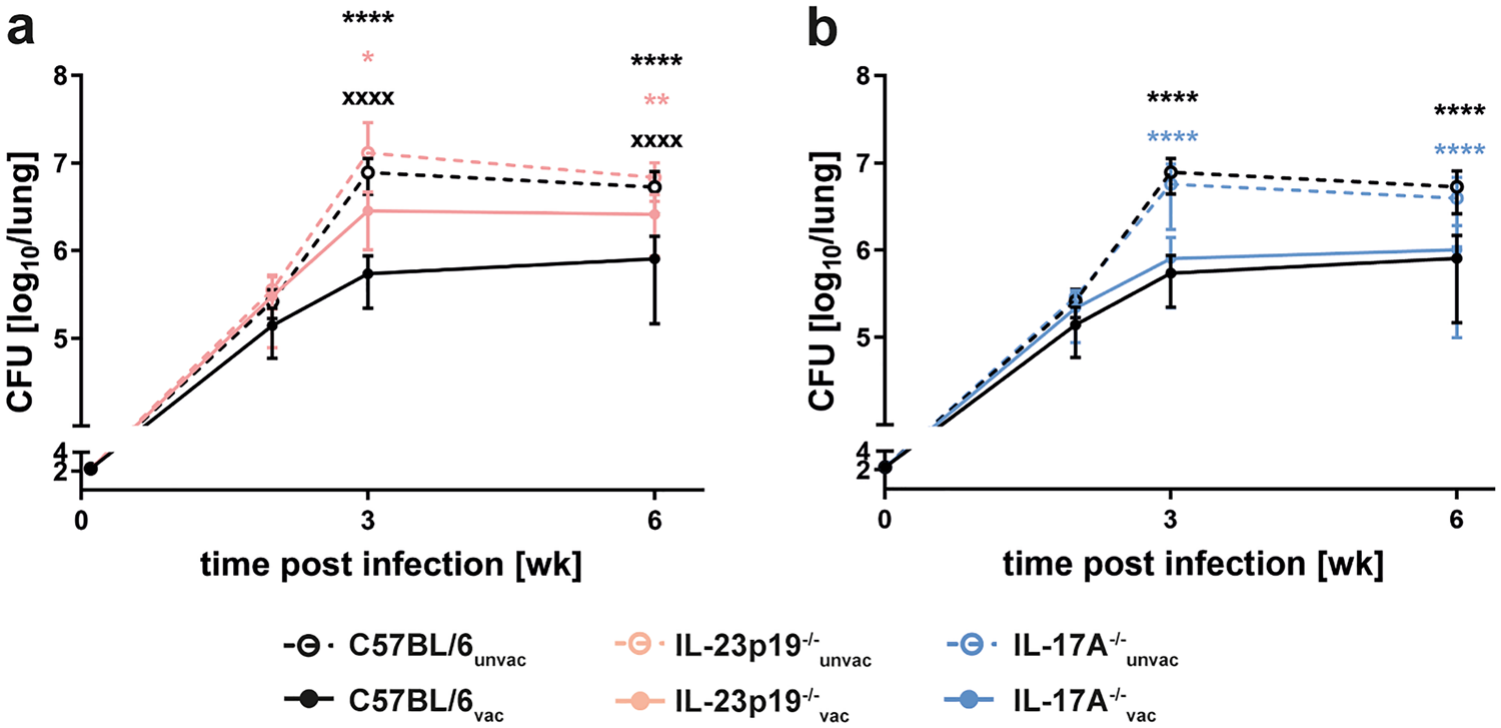
IL-23 contributes to H1-DDA/TDB-induced protection against Mtb infection mostly independent of IL-17A. C57BL/6 (**a** and **b**), IL-23p19^−/−^ (**a**), and IL-17A^−/−^ mice (**b**) were vaccinated via footpad injection of H1 antigen formulated in DDA/TDB three times at 2-week intervals. At the same time control animals were injected with PBS. Four weeks after the third injection, unvaccinated (unvac) and vaccinated (vac) mice were infected with Mtb H37Rv via the aerosol route. At the indicated time points after infection, mycobacterial colony enumeration assays were performed in the lungs. Data represent mean ± SD of 5 mice per group of one experiment (week 2), or 14–15 mice of three (week 3, week 6) independent experiments, respectively. Data for C57BL/6, IL-23p19^−/−^, and IL-17A^−/−^ mice were always obtained in the same experiments, and results of C57BL/6 mice are shown in **a** and **b**. Statistical analysis was performed using a two-way ANOVA with Bonferroni multiple comparison test defining differences between C57BL/6_unvac_ and C57BL/6_vac_ mice, between IL-23p19^−/−^_unvac_ and IL-23p19^−/−^_vac_ mice, and between IL-17A^−/−^_unvac_ and IL-17A^−/−^_vac_ mice (*) as well as between C57BL/6_vac_ and IL-23p19^−/−^_vac_ and between C57BL/6_vac_ and IL-17A^−/−^_vac_ (x) as significant (*p < 0.05, **p < 0.01, ****p < 0.0001, xxxx *p* < 0.0001)

Previous data suggest that the IL-23-IL-17A axis is required for protection against Mtb after vaccination with BCG [30] and after subunit vaccination adjuvanted with TDM [15]. To confirm that IL-23 affects bacterial loads after vaccination with H1-DDA/TDB, we immunized C57BL/6 and IL-23p19^−/−^ mice via footpad injection of H1 antigen formulated in DDA/TDB three times at 2-week intervals. At the same time control animals were injected with PBS. Four weeks after the third injection, unvaccinated and vaccinated mice were infected with approximately 150 CFU Mtb H37Rv via the aerosol route. At the indicated time points after infection, we analyzed bacterial loads in the lungs of unvaccinated and H1-DDA/TDB-vaccinated mice of both strains (Fig. 1a). At week 2 post-infection, bacterial burdens in IL-23p19^−/−^ mice were almost comparable to those in C57BL/6 mice. Here, unvaccinated and vaccinated mice showed similar bacterial burdens in both mouse strains. However, from week 3 post-infection on, as soon as vaccine-induced protection starts in wild-type mice, bacterial burdens in vaccinated IL-23p19^−/−^ mice were significantly higher when compared to those in vaccinated wild-type mice. In contrast, bacterial burdens in the unvaccinated mice of both strains were still comparable. Hence, IL-23 is required for supporting protection against Mtb infection after vaccination with H1-DDA/TDB. IL-17A induced by IL-23 is supposed to promote protection after TDM-adjuvanted subunit vaccination via the chemokine-mediated attraction of Th1 cells to the lung [15]. Moreover, in a mucosal vaccination model IL-17A mediates vaccine-induced protection against Mtb—in this case, however, in an IFN-γ-independent manner [27]. Based on these different findings, we investigated by means of IL-17A^−/−^ mice whether IL-17A is in fact responsible for protection against Mtb after vaccination with H1-DDA/TDB. Therefore, we determined bacterial loads in the lungs of unvaccinated and vaccinated C57BL/6 and IL-17A^−/−^ mice as described above (Fig. 1b). Similar to those in wild-type mice, bacterial burdens in unvaccinated and vaccinated IL-17A^−/−^ mice were not yet significantly different at week 2 post-infection, while vaccination led to significantly reduced bacterial loads at week 3 and 6 post-infection. Together, contrary to expectation, the present study did not identify a continuous correlation between the H1-DDA/TDB-induced Th17 immune response and protection after vaccination. In contrast, protection was generally reduced in the absence of IL-23, confirming the importance of this cytokine for vaccination efficiency against Mtb. Therefore, it is reasonable to infer that IL-23 supports protection after immunization with H1-DDA/TDB also independently of IL-17A.

### IL-17F is upregulated in vaccinated IL-17A^−/−^ mice, but yet dispensable for the vaccine-induced protection against Mtb in the absence of IL-17A

**Fig. 2:**
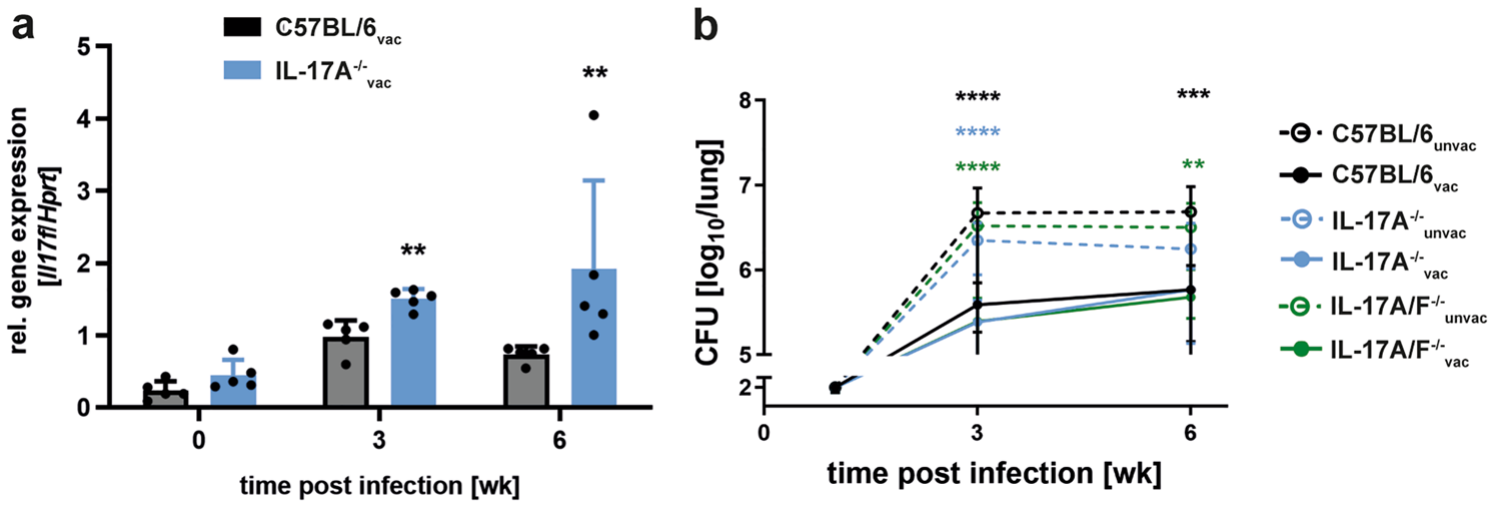
IL17F does not compensate for IL-17A deficiency after vaccination with H1-DDA/TDB in the lungs of Mtb-infected mice. C57BL/6, IL-17A^−/−^, and IL-17A/F^−/−^ mice were vaccinated via footpad injection of H1 antigen formulated in DDA/TDB three times at 2-week intervals. At the same time control animals were injected with PBS. Four weeks after the third injection, unvaccinated (unvac) and vaccinated (vac) mice were infected with Mtb H37Rv via the aerosol route. **a** Gene expression of *Il17f* was quantified by real-time PCR in lung homogenates of vaccinated C57BL/6 and IL-17A^−/−^ mice in uninfected mice and mice infected for 3 and 6 weeks after infection based on the expression of *Hprt*. Data represent mean ± SD of 3–5 mice per group of one experiment representative of two performed. Statistical analysis was performed using a Mann–Whitney test defining differences between C57BL/6_vac_ and IL-17A^−/−^_vac_ mice as significant (**p < 0.01). **b** At the indicated time points after infection, mycobacterial colony enumeration assays were performed in the lungs. Data represent mean ± SD of 9–10 mice per group of two independent experiments. Statistical analysis was performed using a two-way ANOVA with Bonferroni multiple comparison test defining differences between C57BL/6_unvac_ and C57BL/6_vac_ mice, between IL-17A^−/−^_unvac_ and IL-17A^−/−^_vac_ mice, and between IL-17A/F^−/−^_unvac_ and IL-17A/F^−/−^_vac_ mice (*) as well as between C57BL/6_vac_ and IL-17A^−/−^_vac_ and between C57BL/6_vac_ and IL-17A/F^−/−^_vac_ (x) as significant (**p < 0.01, ***p < 0.001, ****p < 0.0001)

The Th17 cytokine IL-17F is structurally closely related to IL-17A [33]. Moreover, both cytokines are located on the same chromosome and signal through the same receptor complex. In the present study, quantitative real-time RT-PCR analysis revealed that after infection with Mtb, the expression of *Il17f* was increased in H1-DDA/TDB-vaccinated IL-17A^−/−^ mice in comparison to vaccinated C57BL/6 mice (Fig. 2a). Therefore, we were wondering whether the elevated levels of IL-17F compensate for the loss of IL-17A and mediate protection after vaccination with H1-DDA/TDB in the absence of IL-17A. To address this question, we compared the outcome of Mtb infection in the lungs of unvaccinated and H1-DDA/TDB-vaccinated mice of the strains C57BL/6, IL-17A^−/−^, and IL-17A/F^−/−^, which lack both IL-17A and IL-17F (Fig. 2b). Mice were thereto vaccinated and infected as described above. Vaccination resulted in significantly lower bacterial burdens in C57BL/6 mice but also in IL-17A^−/−^ and IL-17A/F^−/−^ mice at both examined time points after Mtb infection, indicating that although IL-17F is upregulated in vaccinated IL-17A^−/−^ mice, IL-17F does not compensate for IL-17A deficiency in terms of protection against Mtb infection. Based on this finding, we hereinafter focused on identifying immune mechanisms by which IL-23 mediates vaccine-induced protection against TB in a Th17-independent manner.

### IL-23 and IL-17A promote the vaccine-induced accumulation of antigen-specific Th1 cells in Mtb-infected mice

The unexpected minor role of IL-17A in H1-DDA/TDB-induced protection against Mtb lead us to compare the T cell immune response after vaccination and subsequent Mtb infection in IL-23p19^−/−^, IL-17A^−/−^, and C57BL/6 mice.

**Fig. 3:**
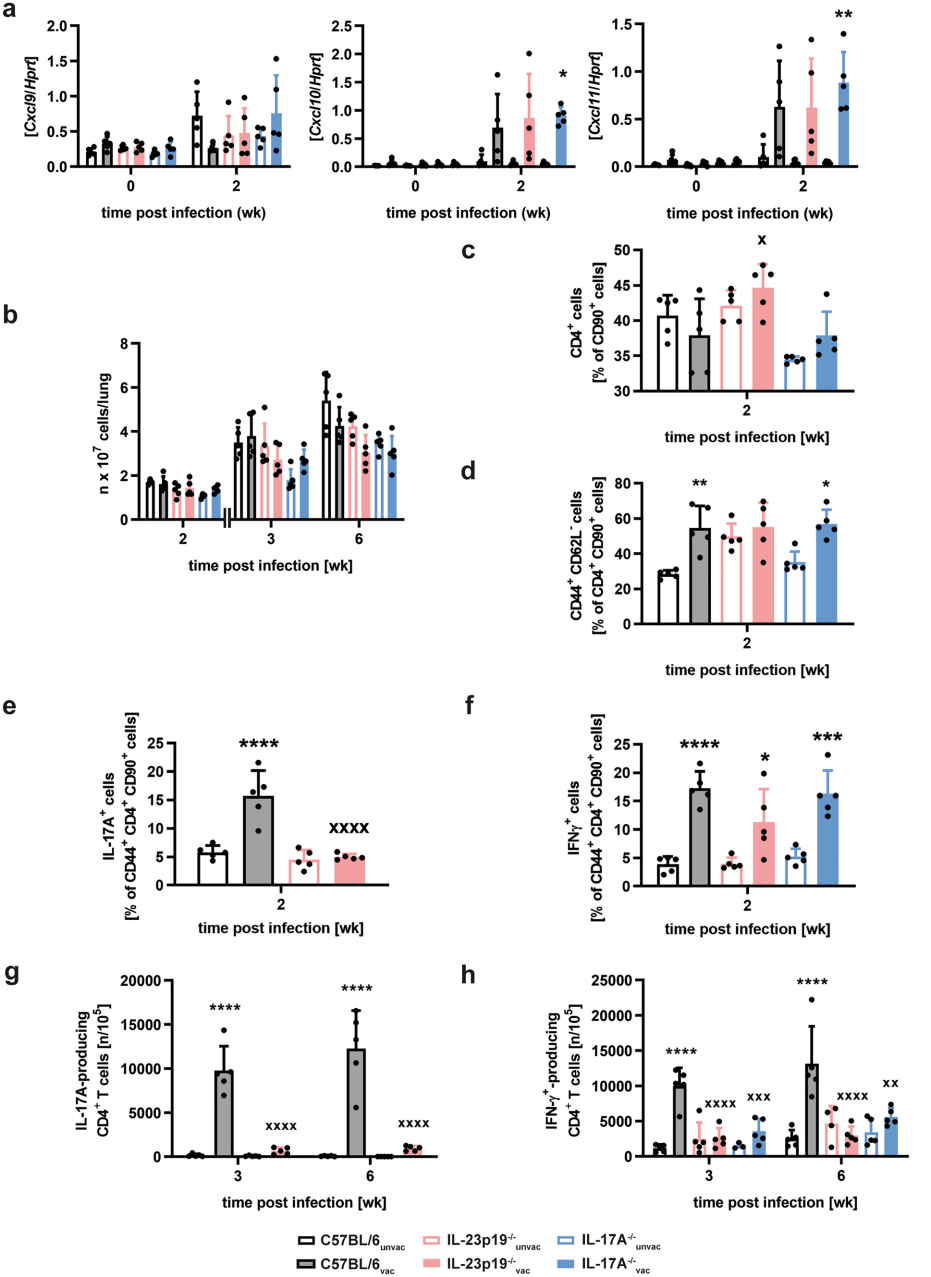
IL23 and IL17A mediate the H1-DDA/TDB-induced accumulation of antigen-specific IFN-γ-producing CD4^+^ T cells in the lungs of Mtb-infected mice. C57BL/6, IL-23p19^−/−^, and IL-17A^−/−^ mice were vaccinated via footpad injection of H1 antigen formulated in DDA/TDB three times at 2-week intervals. At the same time control animals were injected with PBS. Four weeks after the third injection, unvaccinated (unvac) and vaccinated (vac) mice were infected with Mtb H37Rv via the aerosol route. **a** Gene expression of *Cxcl9*, *Cxcl10*, and *Cxcl11* was quantified by real-time PCR in lung homogenates in uninfected mice and mice infected for 2 weeks based on the expression of *Hprt*. Data represent mean ± SD of 5 mice per group of one experiment. **b** Absolute cell numbers were determined at the indicated time points after infection. Data represent mean ± SD of 5 mice per group of one experiment representative of two performed. **c**–**f** Two weeks after infection, lung cells were phenotypically analyzed via flow cytometry. Furthermore, the intracellular cytokine production was analyzed after restimulation with anti-CD3/CD28. The used gating strategy is illustrated in supplemental Fig. S1a. Frequencies of CD4^+^ cells out of CD90^+^ cells (**c**), of CD44^+^ CD62L^−^ out of CD4^+^ CD90^+^ cells (**d**), and frequencies of IL-17A- (**e**) and IFN-γ-producing cells out of CD44^+^ CD4^+^ CD90^+^ cells (**f**) are shown. Data represent mean ± SD of 5 mice per group of one experiment (**c** and **d**) or of one experiment representative of two performed (**e** and **f**). **g** and **h** The frequencies of Esat-6_1–20_-specific IL-17A- (**g**) and IFN-γ-producing cells (**h**) in lung cell suspensions enriched for CD4^+^ T cells were determined by ELISPOT assay in mice infected for 3 and 6 weeks. Data represent mean ± SD of 3–5 mice per group of one experiment representative of two performed. **c**–**h** Data for C57BL/6, IL-23p19^−/−^, and IL-17A^−/−^ mice were always obtained in the same experiments, and results of C57BL/6 mice are also shown in supplemental Fig. S1. Statistical analysis was performed using a two-way ANOVA with Bonferroni multiple comparison test defining differences between C57BL/6_unvac_ and C57BL/6_vac_ mice, between IL-23p19^−/−^_unvac_ and IL-23p19^−/−^_vac_ mice, and between IL-17A^−/−^_unvac_ and IL-17A^−/−^_vac_ mice (*) as well as between C57BL/6_vac_ and IL-23p19^−/−^_vac_ and between C57BL/6_vac_ and IL-17A^−/−^_vac_ (x) mice as significant (*p < 0.05, **p < 0.01, ***p < 0.001, ****p < 0.0001, x *p* < 0.05, xx *p* < 0.01, xxx *p* < 0.001, xxxx *p* < 0.0001)

After subunit vaccination against Mtb, activated CXCR3^+^CD4^+^ T cells accumulate in the lungs of Mtb-infected mice simultaneously with an induction of the CXCR3 ligand chemokines CXCL9, CXCL10, and CXCL11 in the early phase of Mtb infection [15]. Furthermore, it is supposed that the CXCR3 chemokine induction after vaccination is mediated by IL-17A. Accordingly, we analyzed the expression of the chemokines CXCL9, CXCL10, and CXCL11 in unvaccinated and vaccinated mice of the strains C57BL/6, IL-23p19^−/−^, and IL-17A^−/−^ after infection with Mtb via quantitative real-time RT-PCR analysis (Fig. 3a). Mice were thereto vaccinated and infected as described above. At week 2 post-infection, expression levels of *Cxcl10* and *Cxcl11* tended to be higher in vaccinated C57BL/6 and IL-23p19^−/−^ mice and were even significantly increased in vaccinated IL-17A^−/−^ mice when compared to the respective unvaccinated control group. In contrast, for all analyzed mouse strains no vaccine-induced increase in *Cxcl9* expression was observable at week 2 post-infection. Altogether, after vaccination with H1-DDA/TDB the induction of CXCR3 chemokines turned out to be independent of IL-23 and IL-17A. Given that the early expression of the CXCR3 ligand chemokines CXCL9, CXCL10, and CXCL11 were not affected by the absence of the IL-23-IL-17A axis we next analyzed the quality and quantity of the CD4 T cell response after vaccination.

During the whole investigated course of Mtb infection, absolute cell numbers in the lung did not differ considerably between unvaccinated and H1-DDA/TDB-vaccinated mice of all analyzed strains (Fig. 3b). Additionally, no significant variations in cell numbers were detected between vaccinated C57BL/6, IL-23p19^−/−^, and IL-17A^−/−^ mice. In wild-type mice, the overall frequency of CD4^+^ T cells out of CD90^+^ cells was unaffected by the vaccination in the early phase of infection with Mtb, whereas a higher proportion of CD4^+^ T cells had an activation phenotype in vaccinated mice (Fig. S1a, b). Similar to C57BL/6 mice, unvaccinated and H1-DDA/TDB-vaccinated IL-23p19^−/−^ and IL-17A^−/−^ mice exhibited comparable percentages of CD4^+^ T cells in the lung at week 2 post-Mtb infection (Fig. 3c). In contrast, the frequency of CD44^+^CD62L^−^ cells within the CD4^+^CD90^+^ population was significantly enhanced in wild-type and IL-17A^−/−^ mice after vaccination (Fig. 3d). Although no significant difference was detected between unvaccinated and vaccinated IL-23p19^−/−^ mice, vaccinated mice of all strains exhibited comparable percentages of CD44^+^CD62L^−^ cells (Fig. 3d). Thus, the H1-DDA/TDB-induced accumulation of activated CD4^+^ T cells during the early phase of infection with Mtb does not seem to be dependent on the expression of IL-23 and IL-17A. To subsequently analyze Th17 and Th1 immune responses in unvaccinated and vaccinated mice, lung cells were restimulated with anti-CD3/CD28 and the relative amount of IL-17A and IFN-γ-producing cells within the CD44^+^CD4^+^CD90^+^ population was determined by flow cytometry (Fig. S1a, c). Percentages of both IL-17A and IFN-γ-producing activated CD4^+^ T cells were already significantly increased in vaccinated animals in the early phase of Mtb infection, even prior to the establishment of vaccine-induced protection (Fig. S1c). Flow cytometric analysis after polyclonal stimulation with anti-CD3/CD28 showed that the H1-DDA/TDB-induced accumulation of IL-17A-producing cells within the CD44^+^CD4^+^CD90^+^ population at week 2 post-infection was highly impaired in IL-23p19^−/−^ mice (Fig. 3e). As the IL-23-IL-17A axis induced by subunit vaccination is supposed to mediate protection through chemokine-mediated accumulation of Th1 cells in the lung [15], we wondered whether the Th1 induction after vaccination with H1-DDA/TDB is facilitated by IL-23 and IL-17A. Flow cytometric analysis after polyclonal stimulation with anti-CD3/CD28 revealed increased frequencies of IFN-γ-producing cells within the CD44^+^CD4^+^CD90^+^ population after vaccination in C57BL/6, IL-23p19^−/−^, and IL-17A^−/−^ mice (Fig. 3f). Although in IL-23p19^−/−^ mice, the vaccine-induced accumulation of those polyclonal IFN-γ-producing CD4^+^ T cells appeared to be less efficient, no significant differences were detected between vaccinated mice of all three strains (Fig. 3f). Together, early after Mtb infection the chemokine-mediated infiltration of Th1 cells in H1-DDA/TDB-vaccinated mice is not dependent on the IL-23-induced expression of IL-17A. To follow the subsequent antigen-specific Th17 and Th1 immune response after vaccination, ELISPOT assays of purified CD4^+^ T cells after ESAT6_1–20_ restimulation were performed at different time points of infection (Fig. 3g, h). Different from vaccinated C57BL/6 mice, vaccinated IL-23p19^−/−^ mice did not mount an ESAT6_1–20_-specific Th17 immune response in the lung at weeks 3 and 6 post-infection (Fig. 3g). However, the induction of ESAT6_1–20_-specific IFN-γ-producing CD4^+^ T cells after vaccination with H1-DDA/TDB was abrogated in IL-17A^−/−^ and IL-23p19^−/−^ mice (Fig. 3h). Together, these findings indicate that although IL-23 and IL-17A do not affect the polyclonal Th1 immune response, the H1-DDA/TDB-induced accumulation of antigen-specific Th1 cells in Mtb-infected mice is dependent on both IL-23 and IL-17A.

### IL-23 promotes the vaccine-induced accumulation of effector memory T cells in Mtb-infected mice independently of IL-17A

**Fig. 4:**
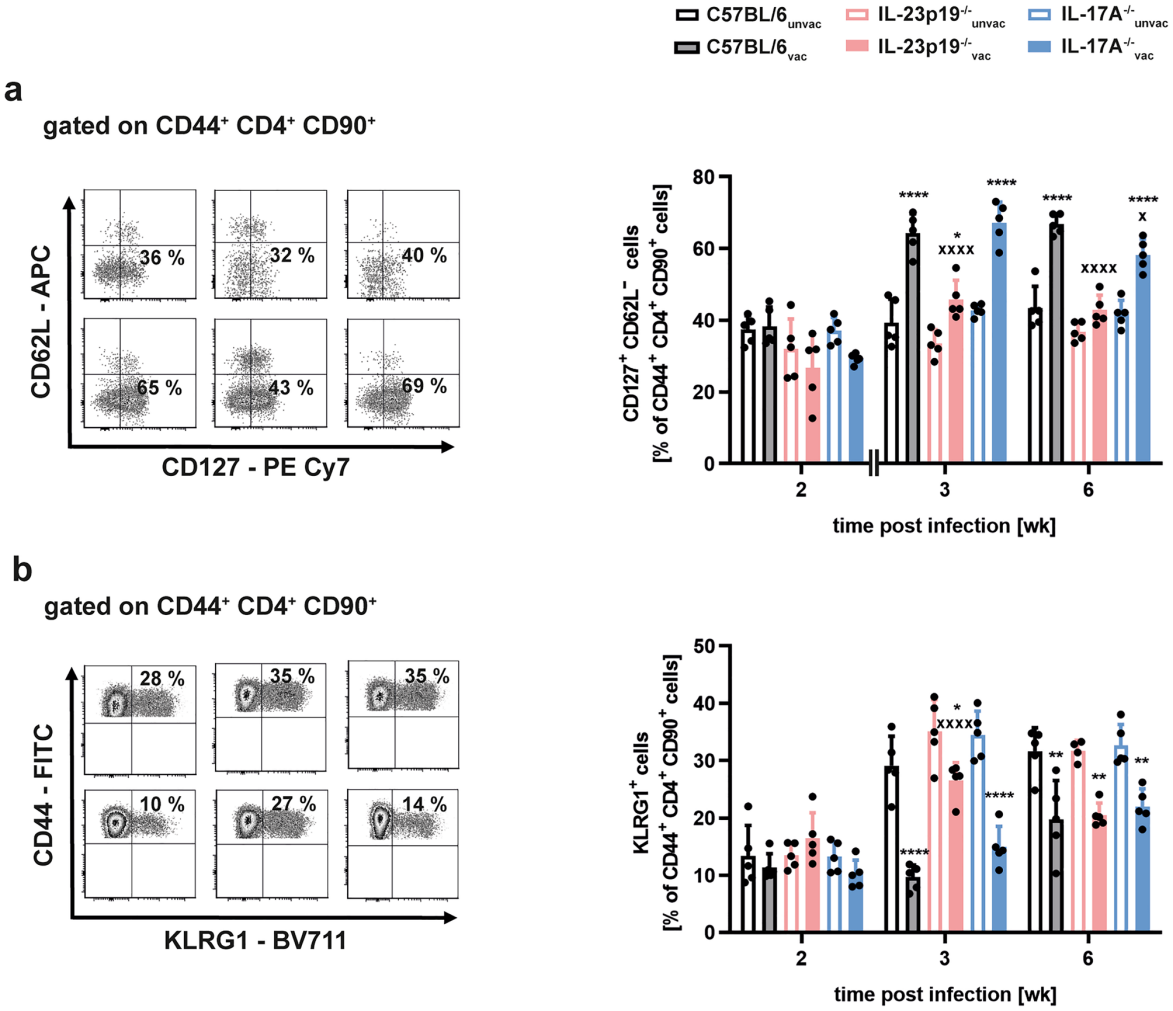
IL-23 but not IL-17A stimulates the H1-DDA/TDB-induced accumulation of effector memory T cells. C57BL/6, IL-23p19^−/−^, and IL-17A^−/−^ mice were vaccinated via footpad injection of H1 antigen formulated in DDA/TDB three times at 2-week intervals. At the same time control animals were injected with PBS. Four weeks after the third injection, unvaccinated (unvac) and vaccinated (vac) mice were infected with Mtb H37Rv via the aerosol route. Lung cells were phenotypically analyzed via flow cytometry. **a** Representative density plots of CD127^+^ CD62L^−^ cells out of CD44^+^ CD4^+^ CD90^+^ cells are shown of week 3 post-infection (left) and frequencies are shown at the indicated time points after infection (right). Data represent mean ± SD of 5 mice per group of one experiment (week 2) or of one experiment representative of two performed (week 3, week 6), respectively. **b** Representative density plots of KLRG1^+^ cells out of CD44^+^ CD4^+^ CD90^+^ cells are shown of week 3 post-infection (left) and frequencies are shown at the indicated time points after infection (right). Data represent mean ± SD of 4–5 mice per group of one experiment (week 2, week 6) or of one experiment representative of two performed (week 3), respectively. **a** and **b** Statistical analysis was performed using a two-way ANOVA with Bonferroni multiple comparison test defining differences between C57BL/6_unvac_ and C57BL/6_vac_ mice, between IL-23p19^−/−^_unvac_ and IL-23p19^−/−^_vac_ mice, and between IL-17A^−/−^_unvac_ and IL-17A^−/−^_vac_ mice (*) as well as between C57BL/6_vac_ and IL-23p19^−/−^_vac_ and between C57BL/6_vac_ and IL-17A^−/−^_vac_ (x) mice as significant (*p < 0.05, **p < 0.01, ****p < 0.0001, x *p* < 0.05, xxxx *p* < 0.0001)

So far, we have shown that IL-23—although facilitating a strong TH17 immune response—supports protection after vaccination with H1-DDA/TDB independently of IL-17A. The vaccine-induced accumulation of antigen-specific Th1 cells on the other hand was observed to be both IL-23- and IL-17A-dependent. This indicates that the magnitude of these cells does not provide a suitable correlate of protection after vaccination. Based on these results, we further asked how IL-23 generates protection after vaccination independently of IL-17A. As IL-23 preferentially acts on CD4^+^ T cells with a memory phenotype [17, 20], we examined whether IL-23 might have a specific role for the induction of memory immune responses after vaccination against Mtb. By flow cytometry, we therefore analyzed the relative amount of CD127^+^CD62L^−^ effector memory T cells within the CD44^+^CD4^+^CD90^+^ population in the lungs of unvaccinated and H1-DDA/TDB-vaccinated, Mtb-infected C57BL/6, IL-23p19^−/−^, and IL-17A^−/−^ mice (Fig. 4a). Mice were thereto vaccinated and infected as described above. In C57BL/6 mice, vaccination increased the percentage of effector memory CD4 T cells at weeks 3 and 6 post-infection. Similarly, also vaccinated IL-17A^−/−^ mice showed elevated percentages of these cells when compared to unvaccinated IL-17A^−/−^ mice. In contrast, in IL-23p19^−/−^ mice vaccination caused only a weak effector memory CD4 T cell induction at week 3 post-infection, with no significant difference between unvaccinated and vaccinated mice observable at week 6 post-infection. In addition, when compared to vaccinated C57BL/6 mice, percentages of effector memory T cells in vaccinated IL-23p19^−/−^ mice were significantly lower (Fig. 4a). Hence, these data suggest that IL-23 indeed modulates the vaccine-induced effector memory CD4 T cell response without any involvement of IL-17A. Alongside with the induction of memory immune responses, prime-boost vaccination with BCG and H1-DDA/TDB was shown to reduce the frequency of effector T cells, which are positive for the inhibitory receptor KLRG1 [13]. During experimental TB these KLRG1^+^CD4^+^ T cells represent a population of terminally differentiated cells with a short life span and a low proliferative capacity [36]. To check whether IL-23 affects the proportion of KLRG1^+^ effector T cells after vaccination with H1-DDA/TDB and Mtb challenge, we analyzed percentages of KLRG1^+^ cells within the CD44^+^CD4^+^CD90^+^ population in unvaccinated and vaccinated mice of the strains C57BL/6, IL-23p19^−/−^, and IL-17A^−/−^ after infection with Mtb (Fig. 4b). While we did not observe major differences between the analyzed mouse groups at week 2 post-infection, vaccination reduced the frequency of KLRG1^+^ effector T cells in all mice after 3 weeks post-infection. However, frequencies of these cells were still significantly higher in vaccinated IL-23p19^−/−^ when compared to vaccinated C57BL/6 mice. At week 6 post-infection, percentages of KLRG1^+^ T cells were decreased in vaccinated C57BL/6, IL-23p19^−/−^, and IL-17A^−/−^ mice to a similar extent. Altogether, IL-23 appears to shift the CD4 T cell immune response after vaccination toward the memory phenotype via an IL*-*17A*-*independent mechanism.

### IL-23 promotes the vaccine-induced accumulation of multifunctional T cells in Mtb-infected mice independently of IL-17A

**Fig. 5:**
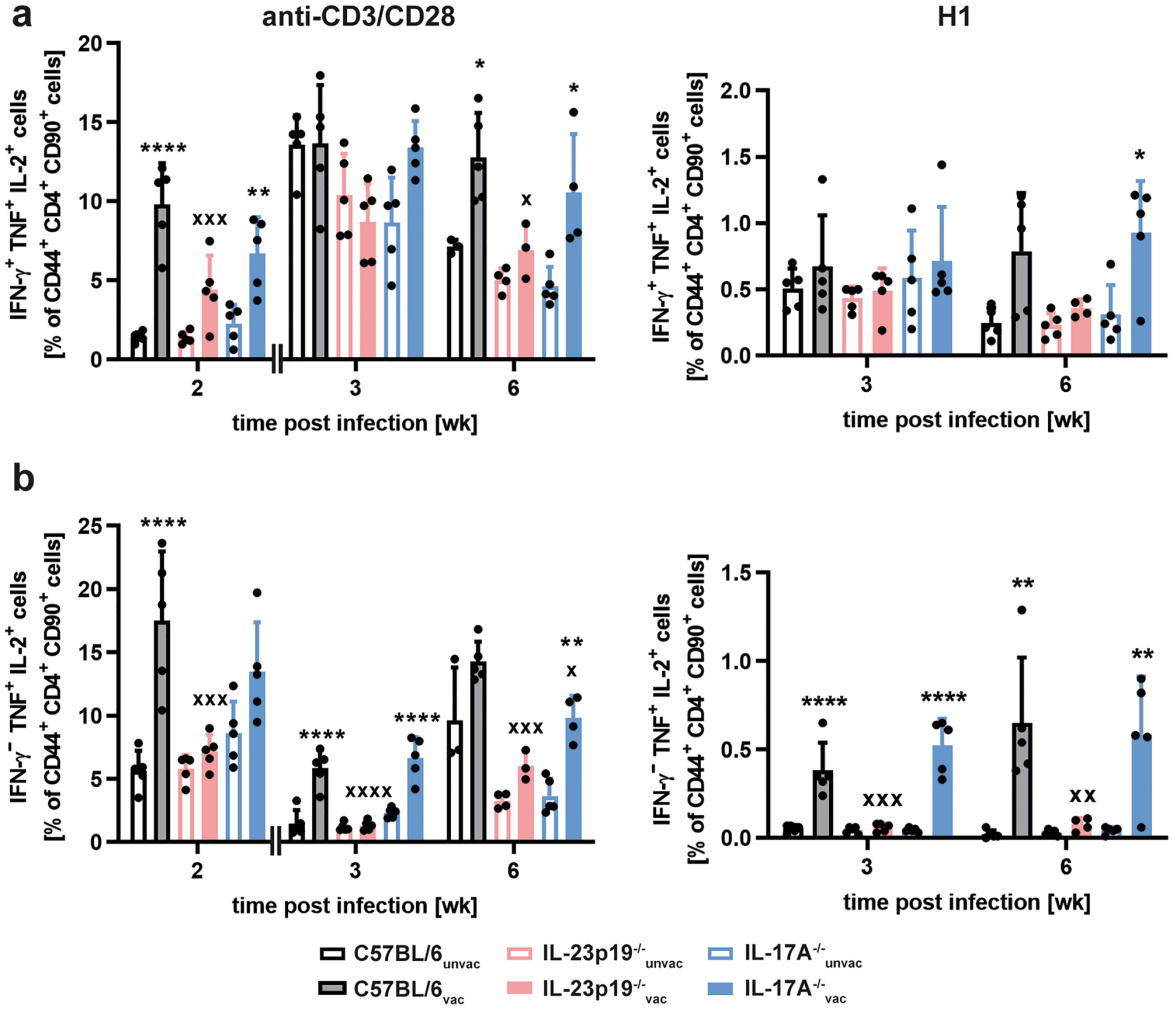
IL-23 but not IL-17A promotes the H1-DDA/TDB-induced accumulation of IFN-γ^−^ TNF^+^ IL-2^+^ double-positive multifunctional CD4 T cells in the lungs of Mtb-infected mice. C57BL/6, IL-23p19^−/−^, and IL-17A^−/−^ mice were vaccinated via footpad injection of H1 antigen formulated in DDA/TDB three times at 2-week intervals. At the same time control animals were injected with PBS. Four weeks after the third injection, unvaccinated (unvac) and vaccinated (vac) mice were infected with Mtb H37Rv via the aerosol route. Lung cells were restimulated with anti-CD3/CD28 (left) or with H1 (right), and the production of the cytokines IFN-γ, TNF, and IL-2 in CD44^+^ CD4^+^ CD90^+^ cells was analyzed via flow cytometry. The used gating strategy is illustrated in supplemental Fig. S2a. At the indicated time points after infection, frequencies of the IFN-γ^+^ TNF^+^ IL-2^+^ triple-positive (**a**) and of the IFN-γ^−^ TNF^+^ IL-2^+^ double-positive (**b**) subpopulation out of CD44^+^ CD4^+^ CD90^+^ cells are shown. Data obtained from anti-CD3/CD28-restimulated mice represent mean ± SD of 3–5 mice per group of one experiment (week 2) or of one experiment representative of two performed (week 3, week 6), respectively. Data obtained from H1-restimulated mice represent mean ± SD of 4–5 mice per group of one experiment. Statistical analysis was performed using a two-way ANOVA with Bonferroni multiple comparison test defining differences between C57BL/6_unvac_ and C57BL/6_vac_ mice, between IL-23p19^−/−^_unvac_ and IL-23p19^−/−^_vac_ mice, and between IL-17A^−/−^_unvac_ and IL-17A^−/−^_vac_ mice (*) as well as between C57BL/6_vac_ and IL-23p19^−/−^_vac_ and between C57BL/6_vac_ and IL-17A^−/−^_vac_ (x) mice as significant (*p < 0.05, **p < 0.01, ****p < 0.0001, x *p* < 0.05, xx *p* < 0.01, xxx *p* < 0.001, xxxx *p* < 0.0001)

Long-term immune responses induced by vaccination with H1-DDA/TDB are dominated by IFN-γ^+^TNF^+^IL-2^+^ triple-positive and IFN-γ^−^TNF^+^IL-2^+^ double-positive multifunctional CD4 T cells [12]. These memory T cell subsets are characterized by a high proliferative potential and superior cytokine production profiles. We were therefore interested whether IL-23 promotes the induction of these subsets of multifunctional T cells after vaccination. As basis of this analysis, we first restimulated lung cells of unvaccinated and H1-DDA/TDB-vaccinated C57BL/6 mice with anti-CD3/CD28 and examined co-expression profiles of IFN-γ, TNF, and IL-2 within the cytokine-positive CD44^+^CD4^+^CD90^+^ population in any combination by flow cytometry (Fig. S2a, b). Vaccination and Mtb infection of the mice were thereto performed as described above. At week 2 post-infection, in contrast to unvaccinated mice, vaccinated mice exhibited primarily triple-positive multifunctional T cells, which co-express all three cytokines. In addition, the proportion of the IFN-γ^**−**^TNF^+^IL-2^+^ double-positive subpopulation was elevated in the vaccinated mice. In the unvaccinated animals, on the other hand, IFN-γ^**−**^TNF^+^IL-2^−^ single-positive CD4^+^ T cells formed the largest proportion. One quality characteristic of multifunctional T cells is the fact that these cells express the highest cytokine amounts on per cell level [12, 37]. This observation was confirmed yet again by our study, as we found that both IFN-γ^**−**^TNF^+^IL-2^+^ double-positive and IFN-γ^+^TNF^+^IL-2^+^ triple-positive multifunctional T cells expressed higher cytokine levels than the single-cytokine-producing CD4^+^ T cell populations (Fig. S2c). Next, we specifically compared the frequencies of IFN-γ^+^TNF^+^IL-2^+^ triple-positive (Fig. 5a) and IFN-γ^**−**^TNF^+^IL-2^+^ double-positive (Fig. 5b) and multifunctional CD4 T cells in the lungs of unvaccinated and vaccinated mice of the strains C57BL/6, IL-23p19^−/−^, and IL-17A^−/−^. After polyclonal restimulation, frequencies of IFN-γ^+^TNF^+^IL-2^+^ triple-positive multifunctional CD4 T cells were enhanced in vaccinated C57BL/6 mice when compared to the unvaccinated C57BL/6 animals at weeks 2 and 6 post-infection (Fig. 5a). Whereas analysis of IL-17A^−/−^ mice revealed a similar tendency, in IL-23p19^−/−^ mice vaccination failed to generate a significant induction of triple-positive multifunctional CD4 T cells. To further display the effect of IL-23 on H1-specific multifunctional CD4 T cells, we restimulated lung cells of unvaccinated and H1-DDA/TDB-vaccinated C57BL/6, IL-23p19^−/−^, and IL-17A^−/−^ mice with the subunit vaccine H1 and analyzed the percentages of IFN-γ^+^TNF^+^IL-2^+^ triple-positive multifunctional T cells within the CD44^+^CD4^+^CD90^+^ population in the lungs by flow cytometry (Fig. 5a). In vaccinated C57BL/6, IL-23p19^−/−^, and IL-17A^−/−^ mice, the frequencies of H1-specific IFN-γ^+^TNF^+^IL-2^+^ triple-positive multifunctional CD4 T cell subsets were similar at all analyzed time points after Mtb infection.

Percentages of IFN-γ^**−**^TNF^+^IL-2^+^ double-positive multifunctional CD4 T cells were significantly increased in vaccinated C57BL/6 mice after polyclonal restimulation at weeks 2 and 3 post-infection (Fig. 5b). Similarly, also vaccinated IL-17A^−/−^ mice showed enhanced frequencies of IFN-γ^**−**^TNF^+^IL-2^+^ multifunctional CD4 T cells until week 6 post-infection. In contrast, in IL-23p19^−/−^ mice no differences between the unvaccinated and the vaccinated group were noticed. Hence, our data indicate that IL-23 mediates the induction of IFN-γ^−^TNF^+^IL-2^+^ multifunctional CD4 T cells after vaccination with H1-DDA/TDB independently of IL-17A. At all investigated time points after Mtb infection, H1-DDA/TDB-vaccinated C57BL/6 and IL-17A^−/−^ mice exhibited enhanced frequencies of H1-specific IFN-γ^**−**^TNF^+^IL-2^+^ double-positive multifunctional CD4 T cells in the lungs when compared to the unvaccinated animals (Fig. 5b). Importantly, the vaccine-induced expansion of this T cell subset was strikingly reduced in IL-23p19^−/−^ mice. These data clearly demonstrate that IL-23 arbitrates the induction of H1-specific IFN-γ^**−**^TNF^+^IL-2^+^ double-positive multifunctional CD4 T cells after vaccination.

### Long-term protection against challenge with Mtb depends on IL-17A

**Fig. 6:**
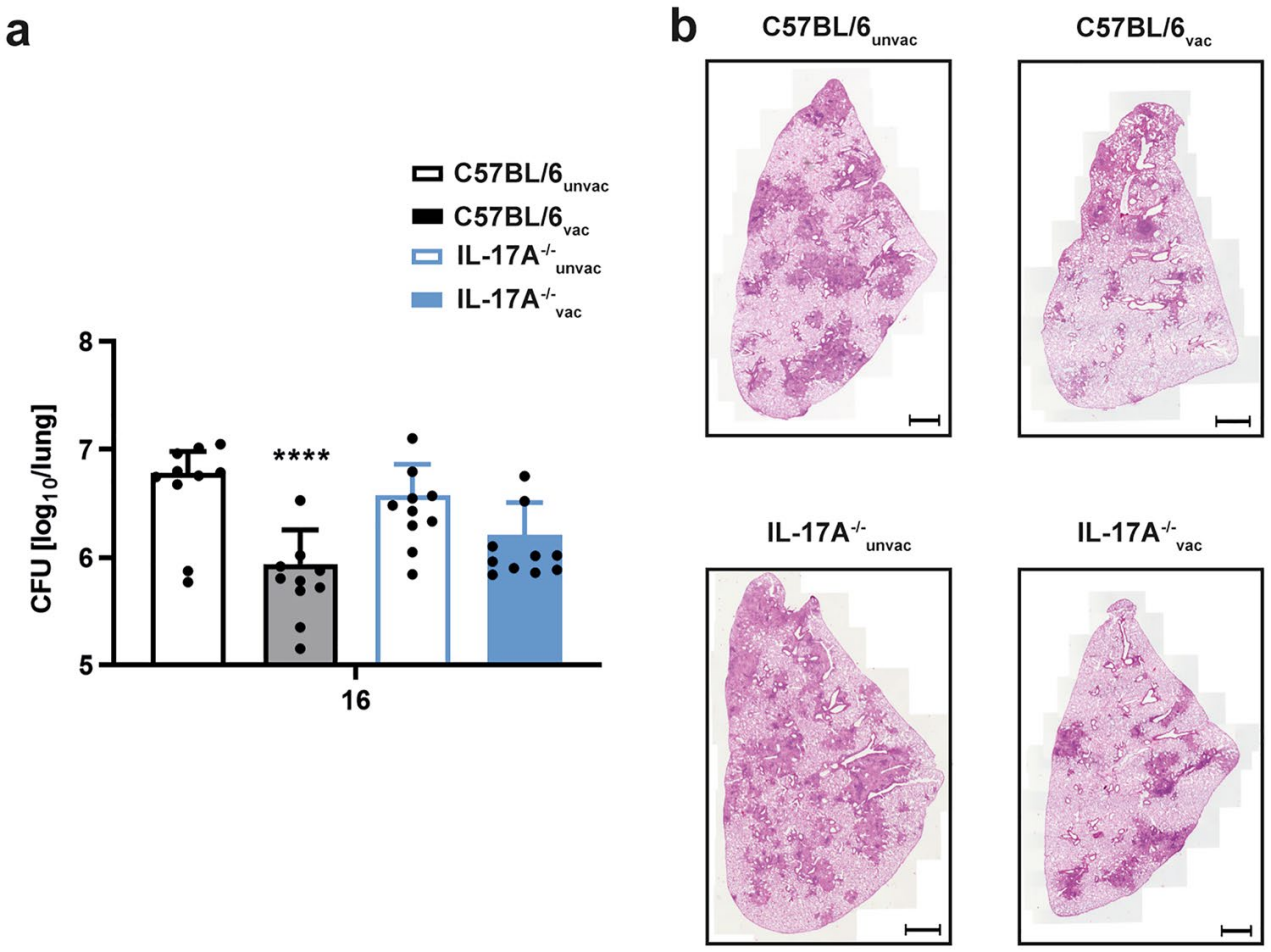
After vaccination with H1-DDA/TDB, IL-17 contributes to long-term protection against Mtb infection. C57BL/6 and IL-17A^−/−^ mice were vaccinated via footpad injection of H1 antigen formulated in DDA/TDB three times at 2-week intervals. At the same time control animals were injected with PBS. Four weeks after the third injection, unvaccinated (unvac) and vaccinated (vac) mice were infected with Mtb H37Rv via the aerosol route. **a** Sixteen weeks after infection, mycobacterial colony enumeration assays were performed in the lungs. Data represent mean ± SD of 10 mice of two independent experiments. Statistical analysis was performed using a two-way ANOVA with Bonferroni multiple comparison test defining differences between C57BL/6_unvac_ and C57BL/6_vac_ mice, between and between IL-17A^−/−^_unvac_ and IL-17A^−/−^_vac_ mice (*), and between C57BL/6_vac_ and IL-17A^−/−^_vac_ (x) as significant (****p < 0.0001). **b** Formalin-fixed lung sections were stained with H&E. Representative sections of experimental mice are shown. Magnification 100 × , bar, 1000 μm

To investigate the extent to which IL-17A is required for long-term vaccination protection against infection with Mtb, C57BL/6 and IL-17A^−/−^ mice were vaccinated and infected with Mtb as described, but the number of CFU in the lungs of these animals was only determined after 16 weeks. In contrast to early time points described so far, protection was still significantly present in vaccinated C57BL/6 mice but appeared to decay in IL-17A^−/−^ animals (Fig. 6a). Although no significant difference could be detected between vaccinated C57BL/6 and IL-17A^−/−^ mice, bacterial burden in vaccinated IL-17A^−/−^ mice was only marginally reduced when compared to unvaccinated IL-17A^−/−^ animals. To examine if the differential bacterial burdens in unvaccinated and H1-DDA/TDB-vaccinated C57BL/6 and IL-17A^−/−^ mice are also reflected by an altered infection-associated lung pathology, we further performed histopathological analysis of lung tissue sections from the experimental mice 16 weeks after Mtb infection (Fig. 6b). Both vaccinated C57BL/6 and IL-17A^−/−^ mice exhibited smaller areas of granulomatous inflammation than the respective unvaccinated control groups. However, lung lesions in vaccinated IL-17A^−/−^ mice appeared to be larger when compared to vaccinated C57BL/6 mice. Together, we conclude that after vaccination IL-23 is required for instructing protection early after Mtb infection independently of IL-17A, whereas IL-17A appears to be required for maintaining long-term protective immune responses.

## Discussion

Over recent years, different animal studies have linked IL-23-dependent Th17 cells to protective immunity after vaccination against experimental TB [15, 23, 24, 27-29, 38]. In this respect, it was assumed that the Th17 signature cytokine IL-17A mediates the vaccine-induced antimicrobial defense through the early induction of chemokines. However, whereas IL-17A turned out to be required for protection in models of mucosal Mtb vaccination [27-29], the role of the cytokine in the context of parenteral vaccination is not yet sufficiently investigated. In the present study, we show that following subunit vaccination with H1-DDA/TDB, IL-17A is—although induced by vaccination during the early phase of Mtb infection—not absolutely required for protection against experimental TB. Instead, we identified IL-23 as a superior mediator of vaccine-induced memory immunity independently of IL-17A.

Augmented expression of IL-17A exhibits a correlate of protection after vaccination with various TB vaccine candidates. Immunization with the recombinant BCG vaccine VPM1002, for instance, induces an enhanced type 17 immune response prior to and in the early phase after Mtb infection, which is accompanied by superior vaccine-induced protection when compared to vaccination with parenteral BCG [39]. Likewise, different types of adjuvants potently augment type 17 immune responses when combined with Mtb antigens. Among these are the glycolipid adjuvants TDM and TDB [15, 23]. In the present study, we confirm the correlation between the induction of a type 17 immune response and protection after vaccination with H1-DDA/TDB. Vaccination here promoted a pronounced pulmonary Th17 immune response, which started early after Mtb infection and preceded the vaccine-induced containment of mycobacterial growth.

Following mucosal vaccination, the expression of IL-17A has also been demonstrated to be causally linked to vaccine-induced protection against Mtb [27-29, 40]. Thus, after mucosal vaccination with ESAT6_1–20_ in combination with LT-IIb, antimycobacterial protection is dependent on IL-17A in an IFN-γ-independent manner [27]. IL-17A thereby promotes the chemokine-mediated strategic positioning of T cells within lung granulomas. Similarly, after pulmonary vaccination with delipidated BCG (dBCG), protection against Mtb also appears to be dependent on Il-17A but does not correlate with an induction of IFN-γ^+^ T cell responses [29]. By contrast, parenteral vaccination with a subunit vaccine adjuvanted with TDM in combination with MPL (monophosphoryl lipid A) and DDA leads to the IL-17A-promoted expression of the CXCR3 ligand chemokines CXCL9, CXCL10, and CXCL11, followed by the recruitment of IFN-γ-producing Th1 cells in the lung, which eventually stop bacterial growth [15]. However, the present study revealed that parenteral vaccination with H1-DDA/TDB generates a similar early reduction of mycobacterial growth in both C57BL/6 and IL-17A^−/−^ mice. Thus, protection after vaccination with H1-DDA/TDB appears not to be completely dependent on IL-17A. In accordance with this finding, vaccination with H1-DDA/TDB promoted an early induction of the chemokines CXCL10 and CXCL11 in the lung, though the deficiency of IL-23 and IL-17A did not have an impact on the expression of these chemokines. Nevertheless, although IL-17A did neither affect the early expression of CXCR3 chemokines nor vaccine-induced protection in the early phase of infection with Mtb, the induction of antigen-specific Th1 cells after vaccination appeared to be dependent on IL-17A. However, the mechanism by which IL-17A mediates the vaccine-induced accumulation of Th1 cells independently of CXCR3 chemokines remains unclear. Surprisingly, while IL-17A has been described to convey protective immune responses after vaccination already in the early phase after challenge with Mtb [15, 27], in the present study, the cytokine turned out to only contribute to long-term protection after vaccination with H1-DDA/TDB. Together, here we could not validate an absolute causal relation between the expression of IL-17A and protection after vaccination. Thus, our findings on parenteral vaccination with H1-DDA/TDB differ from the afore-quoted models of mucosal vaccination [27-29, 40]. In line with recent data on the differences between mucosal and parenteral vaccination in terms of both the vaccine-induced immune response and the vaccination efficiency [41-45], the route of administration might be at least partially responsible for the conflicting results. Moreover, our findings, in juxtaposition with the other aforementioned studies [15, 27, 40], yet again highlight the complex function of IL-17A in the immune response to Mtb. In this context, previous studies have shown that additional factors like the expression strength and the localization of the cytokine may affect the ability of IL-17A to control mycobacterial growth [21, 22, 27]. Furthermore, it is important to consider that the inflammatory cytokine acts as a double-edged sword of immunity, as it mediates protective immune responses to both extracellular and intracellular pathogens [46-50], but also promotes the development of chronic inflammation and autoimmune diseases [51-56]. IL-17A overall seems to play a differential role on the protective immune response to Mtb. Improvement of TB vaccine candidates by inducing IL-17A therefore set specific conditions to ensure both an enhanced efficiency and safety of vaccination.

When assessing the results obtained here, it has to be taken into account that the maintenance of vaccine-induced protection against Mtb in IL-17A^−/−^ mice might result from a compensatory mechanism by other Th17 cytokines. Besides IL-17A, Th17 cells secrete the cytokines IL-17F, IL-21, and IL-22 [16, 57, 58]. IL-17F, just as IL-17A, belongs to the IL-17 family. Both cytokines are encoded by genes located on the same chromosome and are structurally closely related [33]. Moreover, they are recognized by the same receptor [59]. For these reasons, a compensatory effect of IL-17F in the absence of IL-17A would be most likely. In line with this hypothesis, we observed an increased expression of IL-17F in Mtb-infected IL-17A^−/−^ mice when compared to C57BL/6 mice. However, mycobacterial growth was reduced to a similar extent in C57BL/6, IL-17A^−/−^, and in IL-17A/F^−/−^ mice. Thus, in the present study IL-17F appears not to compensate for IL-17A deficiency in IL-17A^−/−^ mice. Possible compensatory mechanisms of IL-21 or IL-22, respectively, were not investigated within the scope of this study.

While we show here that IL-17A is not required for early protection after vaccination with H1-DDA/TDB, our study verifies a major relevance of IL-23 for vaccination against TB. Accordingly, vaccinated IL-23p19^−/−^ mice exhibited higher mycobacterial loads when compared to vaccinated C57BL/6 mice. Based on this finding our aim was to identify IL-23-mediated protective effects, which occur independently of IL-17A.

Initially, IL-23 was described to specifically induce the in vitro proliferation of murine and human memory T cells [17]. In line with this data, in mice the IL-23 receptor subunit IL-23R is almost exclusively expressed on memory T cells [20]. Hence, we were wondering if, after vaccination with H1-DDA/TDB, IL-23 might specifically induce long-lasting memory T cell immune responses. To investigate the vaccine-induced memory T cell response in the lung, at first, we compared the frequency of CD127^+^CD62L^−^ effector memory T cells in C57BL/6, IL-23p19^−/−^, and IL-17A^−/−^ mice. This analysis revealed a vaccine-mediated induction of effector memory T cells which was lost in IL-23p19^−/−^ but not in IL-17A^−/−^ mice. Consequently, IL-23 promotes the effector memory immune response in an IL-17A-independent manner. However, it must be noted that a H1-DDA/TDB-induced effector memory T cell response was not yet detectable in the early phase after infection with Mtb. Future studies might therefore address the impact of IL-23 on other vaccine-induced memory subsets. These might include tissue-resident memory T cells in the lung and central memory T cells, the latter of which were already shown to be of critical importance for protection against Mtb [12]. During experimental TB, the inhibitory receptor KLRG1 identifies a population of short-lived and terminally differentiated effector T cells with a low proliferative potential [13, 36, 60]. In line with our data, it was previously shown that prime-boost vaccination with BCG and H1-DDA/TDB reduced the frequency of KLRG1^+^ CD4 T cells in comparison to vaccination with BCG alone [13]. In contrast to the IL-23-dependent induction of effector memory T cells, our study revealed that overall vaccination with H1-DDA/TDB reduces the proportion of KLRG1^+^ CD4 T cells in the lung independently of IL-17A and IL-23. Altogether, IL-23 supports the H1-DDA/TDB-induced memory T cell immune response. However, while we applied a vaccination and challenge schedule which has been used to initially examine the protective function of IL-23 after immunization against Mtb [15], it would be worthwhile to evaluate the impact of this cytokine on immune memory after vaccination in long-term studies.

As shown in a range of studies, the simultaneous expression of the cytokines IFN-γ, TNF, and IL-2 enables T cells to a more effective immune response towards different infections in mice and men [61-65]. So-called multifunctional T cells appear to be functionally superior when compared to the single cytokine-producing T cell subsets. Accordingly, it was shown in the context of experimental TB that TNF and IFN-γ might act synergistically to induce antimycobacterial activity of macrophages [14, 37, 66]. Co-expression of IL-2 additionally improves the proliferative capacity and long-term survival of the cells [11]. Finally, the quality of multifunctional T cells might also be explained by its high cytokine expression levels at the single-cell level [12, 37, 62]—a finding that was confirmed by the present study. IFN-γ^+^TNF^+^IL-2^+^ triple-positive multifunctional T cells are induced by various Mtb vaccine candidates, among them BCG [12-14, 67, 68]. In contrast, the induction of IFN-γ^−^TNF^+^IL-2^+^ double-positive multifunctional T cells seems to be characteristic of certain subunit vaccines [12, 28]. While triple-positive multifunctional T cells represent an important effector T cell population, IFN-γ^−^TNF^+^IL-2^+^ double-positive multifunctional T cells might build a memory T cell reservoir with a particularly high proliferative potential [12, 37]. After vaccination with H1-DDA/TDB, both IFN-γ^+^TNF^+^IL-2^+^ triple-positive and IFN-γ^−^TNF^+^IL-2^+^ double-positive multifunctional T cells can be detected > 1 year post-vaccination in spleens and peripheral blood and accumulate in the lungs of vaccinated mice after infection with Mtb [35]. Here, we verified the induction of IFN-γ^+^TNF^+^IL-2^+^ triple-positive and IFN-γ^−^TNF^+^IL-2^+^ double-positive multifunctional T cells after vaccination with H1-DDA/TDB and subsequent Mtb infection. Moreover, IL-23, in turn, appeared to be responsible for a selective vaccine-induced expansion of IFN-γ^−^TNF^+^IL-2^+^ double-positive multifunctional T cells in an IL-17A-independent manner. This finding may further confirm the function of IL-23 to directly promote memory T cell responses during Mtb infection after H1-DDA/TDB vaccination.

## Conclusion

In summary, our study validated the importance of IL-23 in protection after vaccination with H1-DDA/TDB against Mtb. However, early antimycobacterial protection was not dependent on the IL-23-mediated expression of IL-17A. Instead, we identified IL-23 as a potential superior mediator of H1-DDA/TDB-induced memory T cell immunity. A systemically elevated expression of IL-23, however, might also cause IL-17A-dependent immunopathology. Hence, a temporally restricted induction of the IL-23-Th17 immune axis during vaccination may minimize such bystander effects. To allow an assessment of a more general function of IL-23 during vaccination, our study has to be extended to different vaccine/adjuvant combinations.

## Supplementary Information

Below is the link to the electronic supplementary material.

Supplementary file1 (DOCX 12211 KB)

## Data Availability

The datasets generated during and/or analyzed during the current study are available from the corresponding author on reasonable request.
