# Supplementary material for: Interleukin-23 instructs protective multifunctional CD4 T cell responses after immunization with the Mycobacterium tuberculosis subunit vaccine H1 DDA/TDB independently of interleukin-17A
Source: J Mol Med (Berl). 2021 Aug 5;99(11):1585–602. doi: 10.1007/s00109-021-02100-3 (PMC8541990; doi:10.1007/s00109-021-02100-3)
Supplement: Supplementary file 1 — Supplementary file1 (DOCX 12211 KB) [file 109_2021_2100_MOESM1_ESM.docx]

**Supplementary information**

**Interleukin-23 instructs protective multifunctional CD4 T cell responses after immunization with the Mycobacterium tuberculosis subunit vaccine H1 DDA/TDB independently of interleukin-17A**

# Kristina Ritter^1^, Jochen Behrends^2^, Hanna Erdmann^1^, Jasmin Rousseau^1,^ Alexandra Hölscher^1^, Johanna Volz^1^, Immo Prinz^3^, Thomas Lindenstrøm^4^, Christoph Hölscher^1^

^1^Infection Immunology, Research Center Borstel, Borstel, Germany; ^2^Fluorescence Cytometry Core Unit, Research Center Borstel, Borstel, Germany; ^3^Institute of Immunology, Hannover Medical School, Hannover, Germany; ^4^Department of Infectious Disease Immunology, Statens Serum Institut, Copenhagen, Denmark

**List of supplementary figures:**

**Fig. S1.** Flowcytometric analysis of antigen-specific IFN-γ- and IL-17A-producing CD4^+^ T cells in the lungs of Mtb infected mice.

**Fig. S2.** Cytokine expression levels of multifunctional CD4 T cells.

**Fig. S3.** Experimental data obtained during repeat experiments.

| 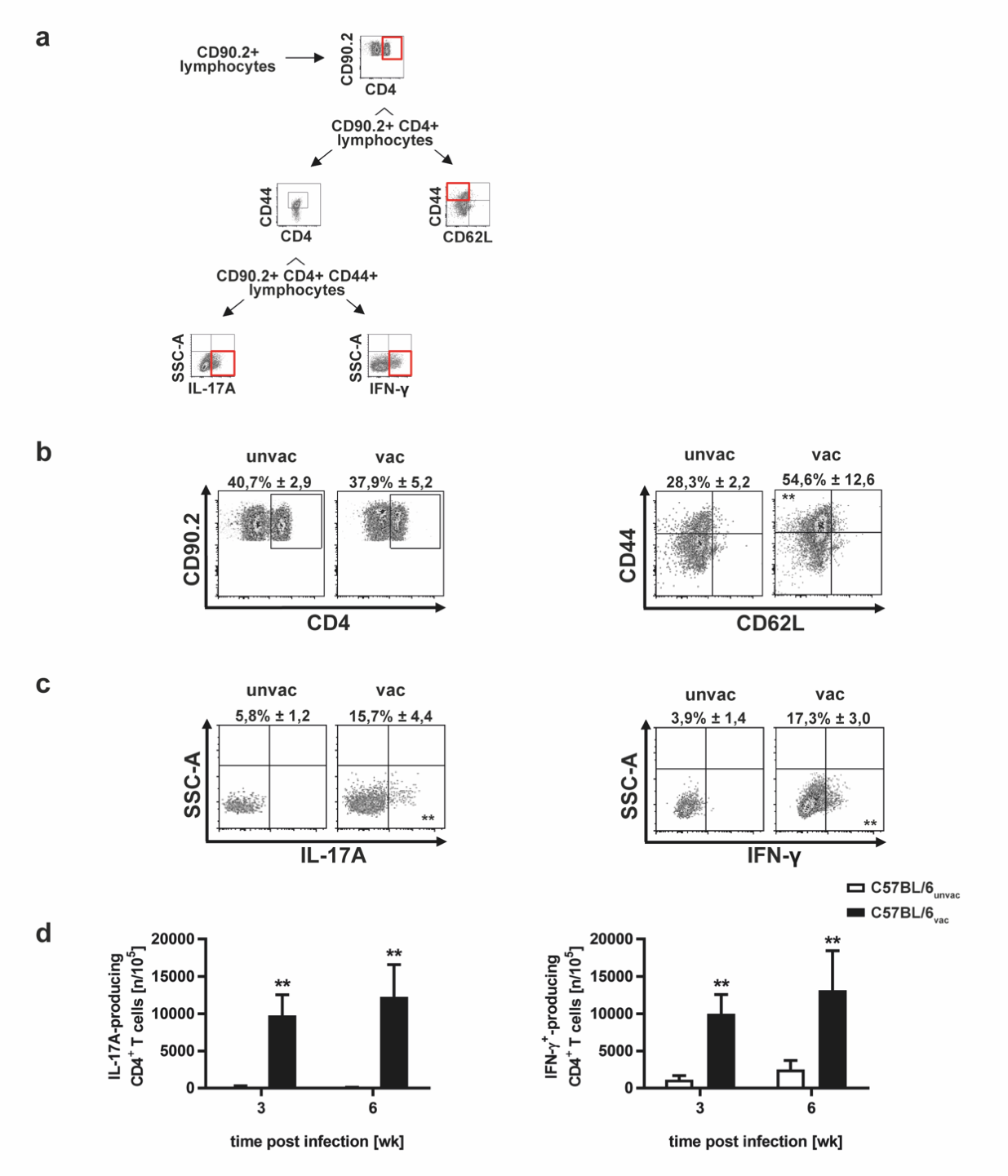 |
| --- |
| **Fig. S1** Vaccination with H1-DDA/TDB triggers the accumulation of antigen-specific IFN-γ- and IL-17A-producing CD4^+^ T cells in the lungs of Mtb infected mice. C57BL/6 mice were vaccinated via footpad injection of H1 antigen formulated in DDA/TDB three times at 2-week intervals. At the same time control animals were injected with PBS. Four weeks after the 3rd injection, unvaccinated (unvac) and vaccinated (vac) mice were infected with Mtb H37Rv via the aerosol route. **a-c** Two weeks after infection, lung cells were phenotypically analyzed via flow cytometry. Furthermore, the intracellular cytokine production was analyzed after restimulation with anti-CD3/CD28. **a** The used gating strategy is illustrated. **b**, **c** Representative density plots and frequencies of CD4^+^ cells out of CD90.2^+^ cells (**b** left), of CD44^+^ CD62L^-^ out of CD4^+^ CD90.2^+^ cells (**b** right) and of IL-17A- (**c** left) and IFN-γ-producing cells (**c** right) out of CD44^+^ CD4^+^ CD90.2^+^ cells are shown. Data represent mean ±SD of 5 mice per group of one experiment (**b**) or of one experiment representative of two performed (**c**) respectively. **d** The frequencies of Esat-6_1–20_ specific IL-17A- (left) and IFN-γ-producing cells (right) in lung cell suspensions enriched for CD4^+^ T cells were determined by ELISPOT assay in mice infected for 3 and 6 weeks. Data represent mean ±SD of 5 mice per group of one experiment representative of two performed. **b-d** Data for C57BL/6, IL-23p19^-/-^, and IL-17A^-/-^ mice were always obtained in the same experiments and results of C57BL/6 mice are also shown in Fig. 3. Statistical analysis was performed using a Mann-Whitney test defining differences between C57BL/6_unvac_ and C57BL/6_vac_ mice as significant (*, p<0.05; **, p<0.01). |

| 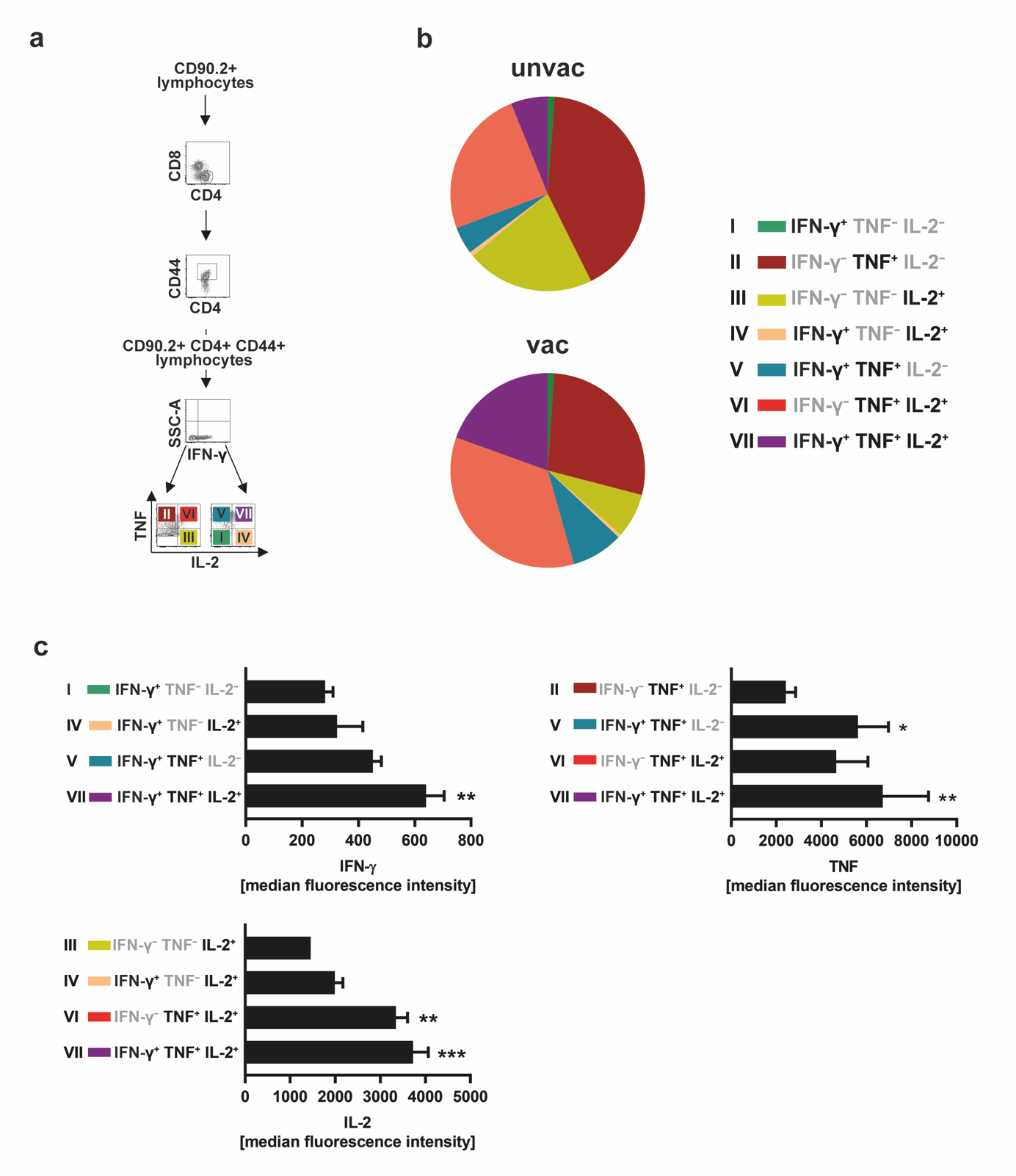 |
| --- |
| **Fig. S2** Multifunctional CD4 T cells display higher cytokine expression levels than the single cytokineproducing CD4^+^ T cell populations. C57BL/6 mice were vaccinated via footpad injection of H1 antigen formulated in DDA/TDB three times at 2-week intervals. Four weeks after the 3rd injection, mice were infected with Mtb H37Rv via the aerosol route. Two weeks after infection, lung cells were restimulated with anti-CD3/CD28 and the production of the cytokines IFN-γ, TNF and IL-2 in CD44^+^ CD4^+^ CD90.2^+^ cells was analyzed via flow cytometry. Based on the different cytokine coexpression profiles 7 subpopulations (I-VII) were built. **a** The used gating strategy is illustrated. **b** For unvaccinated and vaccinated C57BL/6 mice infected for 2 weeks, the relative contributions of each subpopulation to all CD44^+^ CD4^+^ CD90.2^+^ cells are shown in pie charts. Data represent mean ±SD of 5 mice per group of one experiment representative of two performed. **c** the median fluorescence intensity of IFN-γ in the IFN-γ-producing subpopulations, of TNF in the TNF-producing subpopulations and of IL-2 in the IL-2-producing subpopulations was compared. Data represent mean ±SD of 4-5 mice per group of one experiment representative of two performed. Statistical analysis was performed using a Kruskal*-*Wallis test with Dunn‘s multiple comparison test in each case defining differences between single positive and double/triple positive subpopulations mice as significant (*, p<0.05; **, p<0.01; ***, p<0.001). |
| 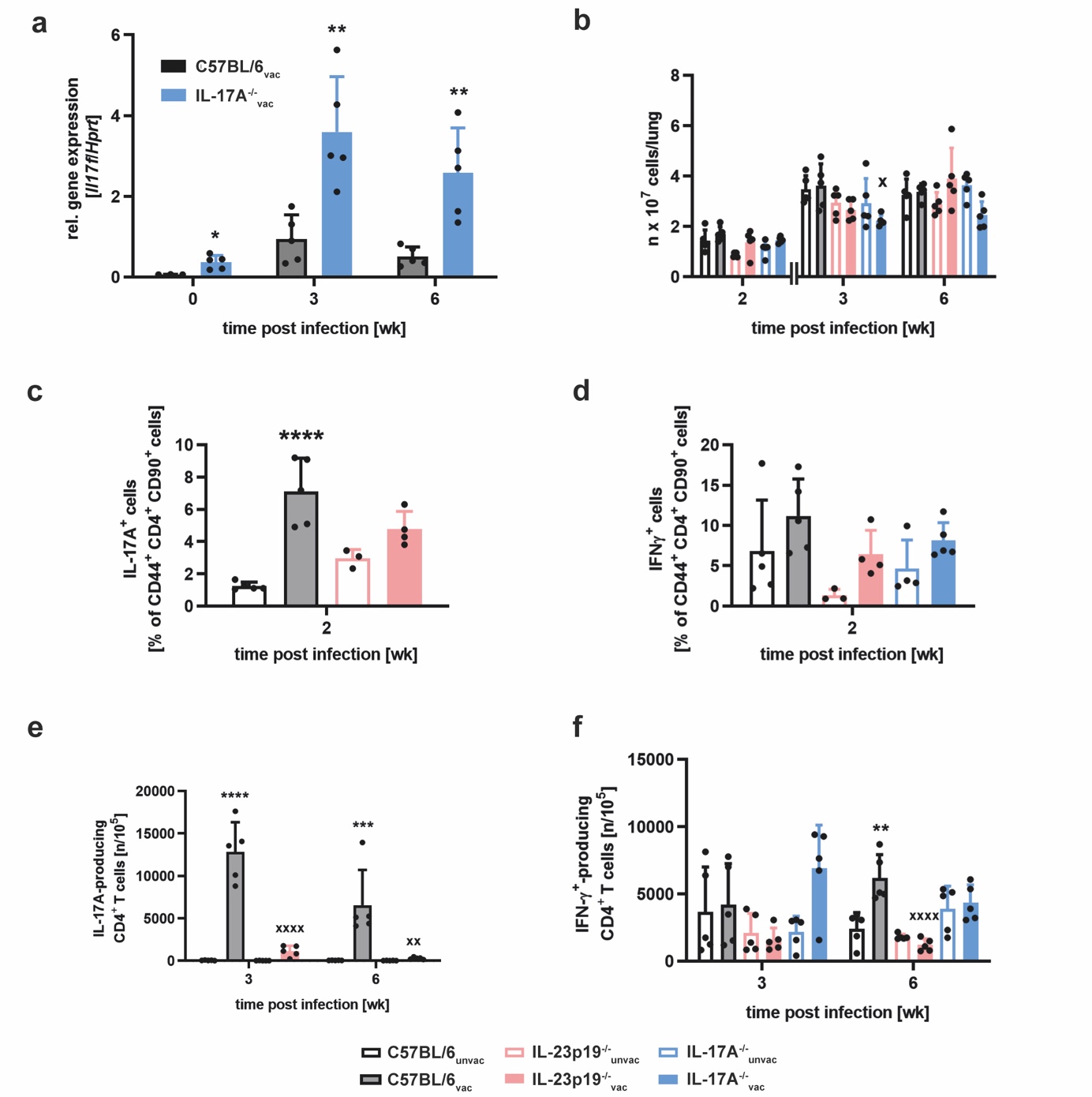 |
| **Fig. S3** Experimental data obtained during repeat experiments. C57BL/6, IL-23p19^-/-^ and IL-17A^-/-^ mice were vaccinated via footpad injection of H1 antigen formulated in DDA/TDB three times at 2-week intervals. At the same time control animals were injected with PBS. Four weeks after the 3rd injection, unvaccinated (unvac) and vaccinated (vac) mice were infected with Mtb H37Rv via the aerosol route. **a** Gene expression of *Il17f* was quantified by real time PCR in lung homogenates of vaccinated C57BL/6 and IL-17A^-/-^ mice in uninfected mice and mice infected for 3 and 6 weeks after infection based on the expression of *Hprt*. Data represent mean ±SD of 3-5 mice per group and constitute an independent repetition of Fig. 2a. Statistical analysis was performed using a Mann-Whitney test defining differences between C57BL/6_vac_ and IL-17A^-/-^_vac_ mice as significant ((*, p<0.05; **, p<0.01). **b** Absolute cell numbers were determined at the indicated time points after infection. Data represent mean ±SD of 5 mice per group and constitute an independent repetition of Fig. 3b. **c-d** Two weeks after infection, lung cells were phenotypically analyzed via flow cytometry. Furthermore, the intracellular cytokine production was analyzed after restimulation with anti-CD3/CD28. The used gating strategy is illustrated in supplemental Fig. 1a. Frequencies of IL-17A- (**c**) and IFN-γ-producing cells out of CD44^+^ CD4^+^ CD90^+^ cells (**d**) are shown. Data represent mean ±SD of 3-5 mice per group and constitute an independent repetition of Fig. 3e and f. **e**, **f** Frequencies of Esat-6_1–20_ specific IL-17A- (**e**) and IFN-γ-producing cells (**f**) in lung cell suspensions enriched for CD4^+^ T cells were determined by ELISPOT assay in mice infected for 3 and 6 weeks. Data represent mean ±SD of 5 mice per group and constitute an independent repetition of Fig. 3g and h. |

| 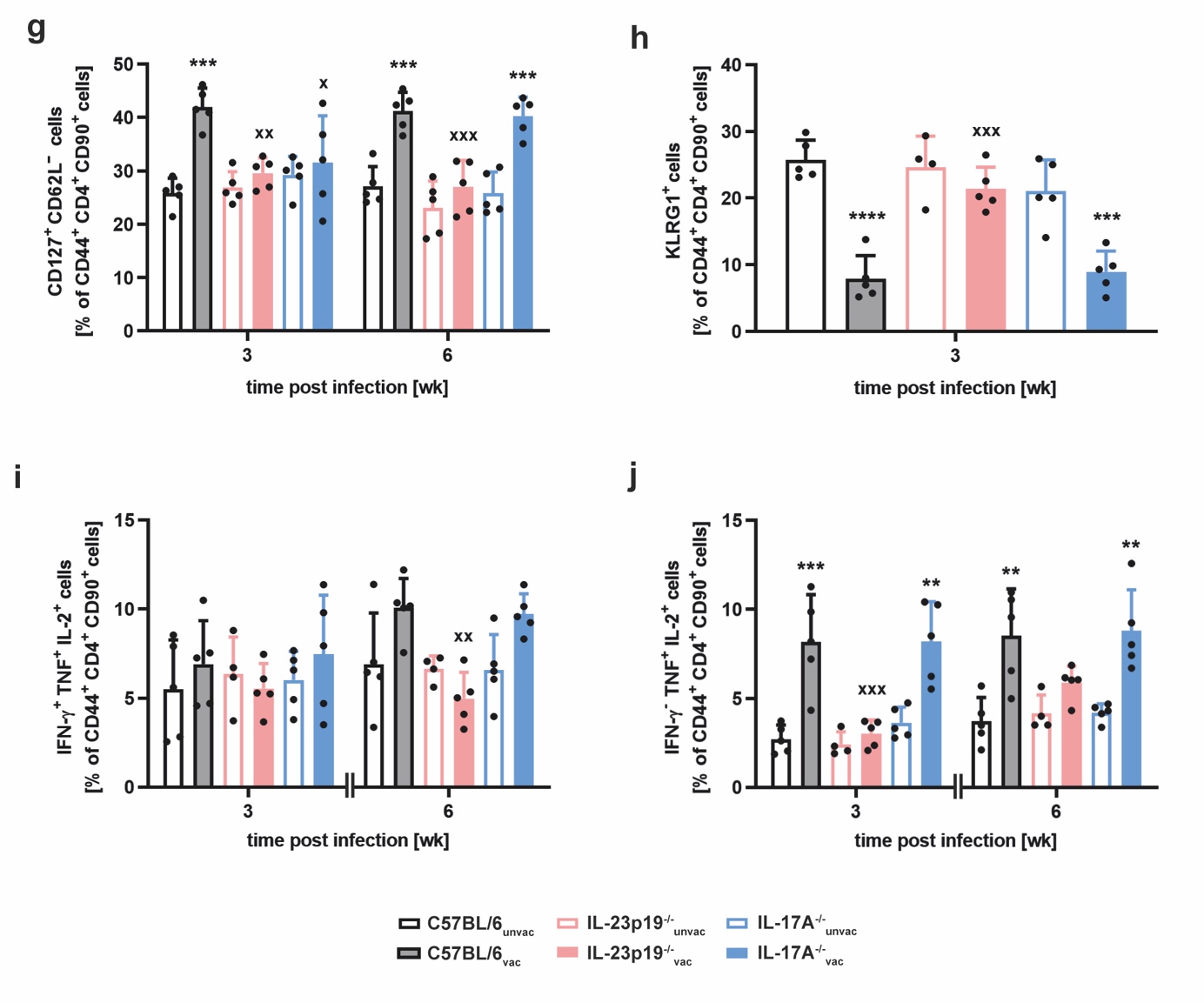 |
| --- |
| **Fig. S3 continued.** Experimental data obtained during repeat experiments. C57BL/6, IL-23p19^-/-^ and IL-17A^-/-^ mice were vaccinated via footpad injection of H1 antigen formulated in DDA/TDB three times at 2-week intervals. At the same time control animals were injected with PBS. Four weeks after the 3rd injection, unvaccinated (unvac) and vaccinated (vac) mice were infected with Mtb H37Rv via the aerosol route. **g-h** Lung cells were phenotypically analyzed via flow cytometry. **g** Frequencies of CD127^+^ CD62L^-^ cells out of CD44^+^ CD4^+^ CD90^+^ cells are shown at the indicated time points after infection. Data represent mean ±SD of 5 mice per group and constitute an independent repetition of Fig. 4a. **h** Frequencies of KLRG1^+^ cells out of CD44^+^ CD4^+^ CD90^+^ cells are shown of week 3 post infection. Data represent mean ±SD of 4-5 mice per group and constitute an independent repetition of Fig. 4b. **i-j** Lung cells were restimulated with anti-CD3/CD28 and the production of the cytokines IFN-γ, TNF and IL-2 in CD44^+^ CD4^+^ CD90^+^ cells was analyzed via flow cytometry. The used gating strategy is illustrated in supplemental Fig. 2a At the indicated time points after infection, frequencies of the IFN-γ^+^ TNF^+^ IL-2^+^ triple positive (**i**) and of the IFN-γ^-^ TNF^+^ IL-2^+^ double positive (**j**) subpopulation out of CD44^+^ CD4^+^ CD90^+^ cells are shown. Mice represent mean ±SD of 4-5 mice per group and constitute an independent repetition of Fig. 5. **b-j** Statistical analysis was performed using a two-way ANOVA with Bonferroni multiple comparison test defining differences between C57BL/6_unvac_ and C57BL/6_vac_ mice, between IL-23p19^-/-^_unvac_ and IL-23p19^-/-^_vac_ mice and between IL-17A^-/-^_unvac_ and IL-17A^-/-^_vac_ mice (*) as well as between C57BL/6_vac_ and IL-23p19^-/-^_vac_ and between C57BL/6_vac_ and IL-17A^-/-^_vac_ (x) mice as significant (**, p<0.01; ***, p<0.001; ****, p<0.0001; x, p<0.05 ; xx, p<0.01; xxx, p<0.001; xxxx, p<0.0001). |
